# Acid ceramidase of macrophages traps herpes simplex virus in multivesicular bodies and protects from severe disease

**DOI:** 10.1038/s41467-020-15072-8

**Published:** 2020-03-12

**Authors:** Judith Lang, Patrick Bohn, Hilal Bhat, Holger Jastrow, Bernd Walkenfort, Feyza Cansiz, Julian Fink, Michael Bauer, Dominik Olszewski, Ana Ramos-Nascimento, Vikas Duhan, Sarah-Kim Friedrich, Katrin Anne Becker, Adalbert Krawczyk, Michael J. Edwards, Andreas Burchert, Magdalena Huber, Justa Friebus-Kardash, Joachim R. Göthert, Cornelia Hardt, Hans Christian Probst, Fabian Schumacher, Karl Köhrer, Burkhard Kleuser, Eduard B. Babiychuk, Beate Sodeik, Jürgen Seibel, Urs F. Greber, Philipp A. Lang, Erich Gulbins, Karl S. Lang

**Affiliations:** 1 Institute of Immunology, University of Duisburg-Essen, Hufelandstr. 55, Essen, D-45147 Germany; 2 Institute of Anatomy, University of Duisburg-Essen, Hufelandstr. 55, Essen, D-45147 Germany; 3 Institut for Experimental Immunology and Imaging, Imaging Center Essen, Electron Microscopy Unit, University of Duisburg-Essen, Hufelandstr. 55, Essen, D-45147 Germany; 4 Institute of Organic Chemistry, Julius-Maximilians University of Würzburg, Am Hubland, Würzburg, D-97074 Germany; 5 Department of Molecular Life Sciences, University of Zurich, Winterthurerstr. 190, CH-8057 Zurich, Switzerland; 6 Institute of Virology, Hannover Medical School, Carl-Neuberg-Str. 1, Hannover, D-30625 Germany; 7 Institute of Molecular Biology, University of Duisburg-Essen, Hufelandstr. 55, Essen, D-45147 Germany; 8 Institute for Virology, University of Duisburg-Essen, Hufelandstr. 55, Essen, D-45147 Germany; 9 Department of Infectious Diseases, University Hospital of Essen, University of Duisburg-Essen, Hufelandstr. 55, Essen, D-45147 Germany; 10 Department of Surgery, University of Cincinnati, Cincinnati, OH USA; 11 Department of Hematology, Oncology and Immunology, University Hospital Giessen and Marburg, Campus Marburg, Baldingerstr., Marburg, D-35043 Germany; 12 Institute of Medical Microbiology and Hospital Hygiene, Philipps-University Marburg, Hans-Meerwein Str. 2, Marburg, D-35043 Germany; 13 Department of Hematology, West German Cancer Center, University Hospital of Essen, University of Duisburg-Essen, Hufelandstr. 55, Essen, D-45147 Germany; 14 Institute of Immunology, University Medical Center Mainz, Langenbeckstr. 1, Mainz, D-55131 Germany; 15 Institute of Nutritional Science, University of Potsdam, Arthur-Scheunert Allee 114-116, Nuthetal, D-14558 Germany; 16 Biological and Medical Research Center (BMFZ), Heinrich-Heine−University, Universitätsstr. 1, Düsseldorf, D-40225 Germany; 17 Institute of Anatomy, University of Bern, Baltzerstr. 4, CH-3012 Bern, Switzerland; 18 Cluster of Excellence RESIST (EXC 2155), Hannover Medical School, Carl-Neuberg-Str. 1, Hannover, D-30625 Germany; 19 Department of Molecular Medicine II, Medical Faculty, Heinrich Heine University, Universitätsstr. 1, Düsseldorf, D-40225 Germany

**Keywords:** Sphingolipids, Membrane fusion, Phagocytosis, Immunology, Infection

## Abstract

Macrophages have important protective functions during infection with herpes simplex virus type 1 (HSV-1). However, molecular mechanisms that restrict viral propagation and protect from severe disease are unclear. Here we show that macrophages take up HSV-1 via endocytosis and transport the virions into multivesicular bodies (MVBs). In MVBs, acid ceramidase (aCDase) converts ceramide into sphingosine and increases the formation of sphingosine-rich intraluminal vesicles (ILVs). Once HSV-1 particles reach MVBs, sphingosine-rich ILVs bind to HSV-1 particles, which restricts fusion with the limiting endosomal membrane and prevents cellular infection. Lack of aCDase in macrophage cultures or in *Asah1*^−/−^ mice results in replication of HSV-1 and *Asah1*^−/−^ mice die soon after systemic or intravaginal inoculation. The treatment of macrophages with sphingosine enhancing compounds blocks HSV-1 propagation, suggesting a therapeutic potential of this pathway. In conclusion, aCDase loads ILVs with sphingosine, which prevents HSV-1 capsids from penetrating into the cytosol.

## Abstract (web summary)

Macrophages are critical in limiting replication of herpes simplex virus type 1 (HSV-1). Here the authors show how acid ceramidase and its enzymatic product sphingosine enable multivesicular bodies to function as an anti-viral mechanism.

## Introduction

Macrophages are one of the most important components of the innate immune system. With their strong phagocytic capacity they engulf pathogens early after their invasion and inactivate them by proteolysis^1^. Especially during virus infection, macrophages are the key effector cells of innate immunity. Mice lacking macrophages are highly susceptible for instance to mouse hepatitis virus, lymphocytic choriomeningitis virus and vesicular stomatitis virus and show overwhelming viral propagation^2–4^. The contribution of macrophages to control of systemic infection was linked to their capacity to internalize infectious particles and limit their propagation in a type I interferon (IFN-I) dependent manner, which leads to upregulation of the antiviral genes Mx, OAS, RNase L and Protein kinase R^4^. While these antiviral mechanisms require hours after infection to become active, macrophages also exhibit robust antiviral suppression already minutes after virus inoculation^4–6^, suggesting the presence of preexisting IFN-I-independent antiviral mechanisms. However, these mechanisms are poorly defined.

Herpes simplex virus type 1 (HSV-1) is an enveloped DNA virus, which can replicate in several different cell types^7^. After binding to a target cell, HSV-1 either fuses directly with the plasma membrane or is taken up into endosomes of the cell^8,9^. Within these endosomes fusion of the outer limiting membrane with HSV-1 membrane leads to infection of the cells in a pH dependent manner^10^. Upon penetration, the HSV-1 capsid traffics on microtubules to the nucleus^11,12^, uncoats its DNA genome at the nuclear pore complex^13,14^, and delivers the genome to the nucleus for replication and assembly of capsids^15,16^. Newly formed capsids bud through the nuclear envelope^17^ and exit the infected cell through the secretory pathway or by cell lysis^18,19^. In an HSV-1 infection model, macrophages play an essential role^20^. This is linked to the high IFN-I sensitivity of HSV-1, which limits the replication of HSV-1 DNA and the production of newly formed capsids^21,22^. Interestingly even in the absence of IFN-I replication of HSV-1 is restricted in macrophages, a phenomenon that could be explained by limited uptake of HSV-1 into macrophages or an aggravated or limited infection of the macrophage after HSV-1 has entered the endosome. Whether indeed macrophage-specific mechanisms regulate HSV-1 infection remains unknown.

Mulitvesicular bodies (MVBs) are complex membrane rich organelles of the cytoplasm, which contain high numbers of vesicles. These so called intraluminal vesicles (ILVs) are formed by invagination and budding from the outer limiting membranes (OLMs) into the MVB lumen and play a crucial role in sorting, degrading, and recycling of proteins^23^. Sphingolipids are enriched in MVBs and are involved in the MVB biogenesis and the regulation of their functions^24^. Thereby sphingolipids modulate diverse cellular processes, including proliferation, differentiation, apoptosis, signal transduction, and membrane trafficking^25–27^. Modulation of biophysical membrane properties such as fluidity, polarity, vesicle formation, and deformation is one major factor of the regulatory capacity of sphingolipids^28^. A key enzyme in sphingolipid metabolism in MVBs is the acid ceramidase (aCDase)^29–31^. It catalyzes the degradation of ceramide into sphingosine and free fatty acid, which modulates the shape and the charge of membranes. Whether this process can modulate HSV-1 entry remains unknown.

Here we analyze whether the aCDase dependent modulation of membrane properties can affect HSV-1 entry after uptake into macrophages. In a model of HSV-1 infection in bone marrow derived murine macrophages (BMDMs) we found that strong activity of aCDase blocks the fusion of the HSV-1 membrane with the outer limiting membrane of MVBs. Antiviral activity of aCDase is independent of IFN-I, but aCDase expression is dependent on IRF8 during macrophage development. In vivo, expression of aCDase in macrophages limits early steps of HSV-1 propagation and protects mice from severe outcome of infection.

## Results

### Macrophages control HSV-1 infection

**Fig. 1:**
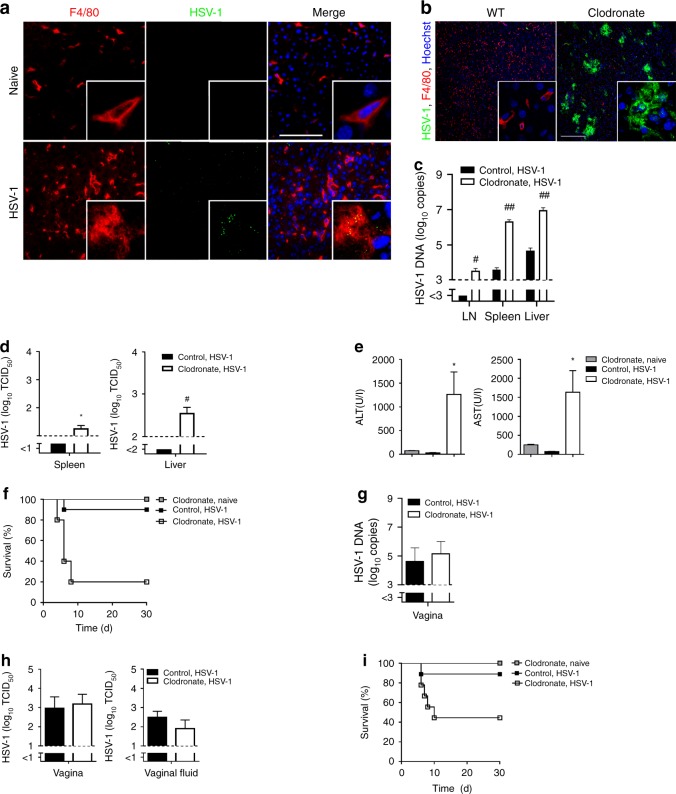
Macrophages control HSV-1 infection. **a** Immunofluorescence of livers from wild-type (WT) mice that were not or were infected with 8 × 10^7^ tissue culture infection dose 50 (TCID_50_) HSV-1 and analyzed after 1 h (*n* = 3, blue represents Hoechst staining, scale bar 100 µm). **b**–**d** Immunofluorescence of livers (**b**, *n* = 8, scale bar 200 µm), real-time polymerase chain reaction (RT-PCR) of lymph nodes (LN), spleens and livers (**c**, *n* = 12–14, two-tailed student’s *t*-test) and TCID_50_ of spleens and livers (**d**, *n* = 8, two-tailed student’s *t*-test) from WT mice that were pretreated with control-liposomes and WT mice that were pretreated with clodronate-liposomes (day −3), infected with 6 × 10^6^ TCID_50_ HSV-1 and analyzed after 24 h. **e**, **f** Alanine transaminase (ALT) and aspartate transaminase (AST) activity (**e**; *n* = 10, one-way ANOVA [Tukey’s multiple comparison]) and survival of mice (**f**) that were treated with clodronate-liposomes or control liposomes (*n* = 10; day -3) and then infected with 6 × 10^5^ TCID_50_ HSV-1 or left uninfected (*n* = 5; *p* = 0.0006, Log-rank [Mantel-Cox] test). **g**, **h** RT-PCR of vagina (**g**) and TCID_50_ of vagina and vaginal fluid (**h**) of mice that were treated with clodronate-liposomes or control-liposomes (day −3) and then infected with 2 × 10^7^ TCID_50_ HSV-1 intravaginally, analyzed after 24 h (*n* = 6, two-tailed student’s *t*-test). **i** Survival of mice that were treated with clodronate-liposomes or control-liposomes (day −3) and then infected with 2 × 10^7^ TCID_50_ HSV-1 intravaginally (*n* = 9), or left uninfected (*n* = 5; *p* = 0.0372, Log-rank [Mantel-Cox] test). All data are shown as mean ± SEM. *p ≤ 0.05, **p ≤ 0.01, ^#^*p* ≤ 0.001, ^##^*p* ≤ 0.0001.

To prove the importance of macrophages during herpes simplex virus type 1 (HSV-1) control in vivo, we infected C57BL/6 mice intravenously with HSV-1 and then analyzed the distribution of virus in the liver by histology. Naïve mice and HSV-1 infected macrophage-depleted mice served as controls. 1 h after infection most of the virus inoculum co-localized with F4/80^+^ macrophages within the liver (Fig. 1a), suggesting that macrophages are equipped to take up HSV-1 quickly after systemic administration. Naïve mice did not show HSV-1 staining (Fig. 1a) and macrophage-depleted mice showed no F4/80^+^ cells in spleen and liver and no co-localization (Supplementary Fig. 1)^4^. To obtain insights on the functional role of macrophages we treated C57BL/6 mice with clodronate-liposomes to deplete macrophages. Control mice were treated with control-liposomes and all mice were infected with HSV-1. In the presence of macrophages, HSV-1 replication was nearly undetectable 24 h after infection (Fig. 1b, c). In strong contrast, HSV-1 showed overwhelming replication in the absence of macrophages (Fig. 1b, c). Enhanced replication of HSV-1 correlated with production of infectious HSV-1 in liver and spleen of macrophage deficient mice (Fig. 1d). In line with overwhelming virus propagation, macrophage deficient mice developed liver cell damage and died quickly after infection (Fig. 1e, f). To analyze the importance of macrophages during a natural infection route, we infected mice intravaginal with HSV-1 and analyzed local replication of HSV-1 and outcome of the infection. Local HSV-1 replication and production of infectious particles was comparable between control and macrophage-depleted mice (Fig. 1g, h). However, macrophage-depleted mice died after intravaginal infection while control mice were protected (Fig. 1i). This suggests that macrophages in the vagina do not limit early local HSV-1 replication, while, macrophages are critical for the systemic control of HSV-1. In conclusion, we found that macrophages do play an essential role to protect from systemic HSV-1 propagation.

### aCDase in multivesicular bodies traps HSV-1

**Fig. 2:**
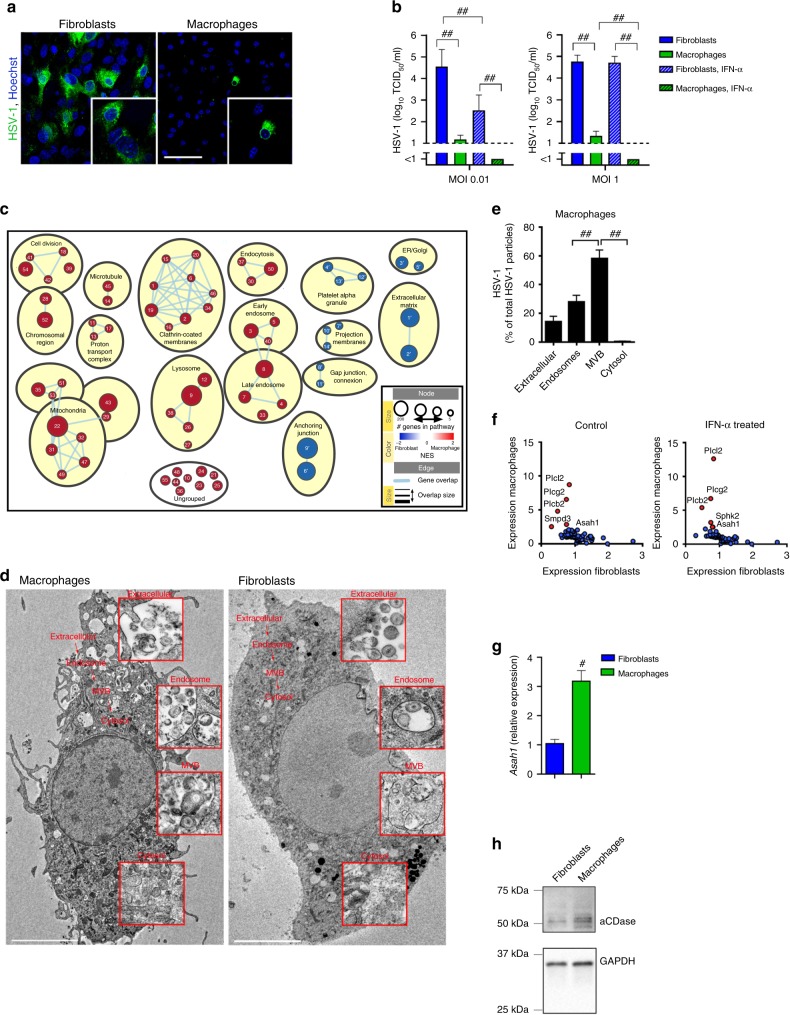
aCDase in multivesicular bodies traps HSV-1. **a**, **b** Immunofluorescence (**a**) and tissue culture infection dose 50 (TCID_50_) from supernatants (**b**) of fibroblasts or bone marrow derived macrophages (BMDMs) that were infected with herpes simplex virus type 1 (HSV-1) at a multiplicity of infection (MOI) of 10 and stained after 6 h (**a**, *n* = 4; scale bar 50 µm) or MOI 0.01 or MOI 1 and supernatant was collected after 24 h (**b**, *n* = 8; one-way ANOVA [Tukey’s multiple comparison]). **c** Gene set enrichment analysis (GSEA) of microarrays showing gene sets enriched in macrophages (red) vs. gene sets enriched in fibroblasts (blue). See Supplementary Table 1a, b for gene sets, ranking and grouping. NES: normalized enrichment score. ER: endoplasmic reticulum. **d**, **e** Representative electron microscopic images (**d**) and quantification (**e**) of wild-type (WT) BMDMs and/or fibroblasts infected with HSV-1 (MOI 250) analyzed after 30 min (BMDMs: *n* = 77 images from three independent experiments, scale bar 5 µm; Fibroblasts: *n* = 106 images from three independent experiments, scale bar 5 µm, one-way ANOVA [Tukey’s multiple comparison]). Overview of one macrophage and one fibroblast is shown. Details show specific compartments containing HSV-1. MVB: multivesicular bodies. **f**: Expression of membrane-modulating proteins analyzed from microarrays of fibroblasts and macrophages with or without 50 U/ml interferon-α4 (IFN-α4) treatment. **g** Real-time polymerase chain reaction (RT-PCR) for *Asah1* mRNA expression of fibroblasts and macrophages from WT mice (*n* = 6, two-tailed student’s *t*-test). **h** Western Blot for aCDase protein expression of fibroblasts and macrophages from WT mice (1 of 2 is shown). All data are shown as mean ± SEM. *p ≤ 0.05, **p ≤ 0.01, ^#^*p* ≤ 0.001, ^##^*p* ≤ 0.0001.

As elucidated in Fig. 1, macrophages are of high importance to limit HSV-1 propagation during systemic infection. Following this, we aimed to investigate antiviral mechanisms that are specific to macrophages. To get insights into the macrophage-specific antiviral mechanism we generated bone marrow derived macrophages (BMDMs) and compared their antiviral activity to isolated alveolar fibroblasts. HSV-1 propagated strongly in fibroblasts and infectious HSV-1 was released into the supernatant (Fig. 2a, b). In contrast to fibroblasts, HSV-1 showed limited propagation in macrophages and infectious particles were hardly measurable in the supernatants (Fig. 2a, b). Even higher infection doses up to MOI 1 did not result in productive infection in cultured macrophages (Fig. 2b). Treatment with interferon-α4 (IFN-α4) reduced the infectious particles (Fig. 2b), however the striking difference in virus production between fibroblasts and macrophages remained. These results suggest that administration of type I interferon (IFN-I) cannot induce the strong antiviral capacities of macrophages in fibroblasts. While there are multiple differences between macrophages and fibroblasts, we hypothesized that macrophages have specific antiviral protection mechanisms independent of the presence of IFN-I.

To explore potential antiviral mechanisms we performed microarray analysis to compare cellular pathways between macrophages and fibroblasts. The strongest difference between fibroblasts and macrophages was the high expression of endosomal, lysosomal and vesicular transport pathways in macrophages (Fig. 2c). From these data, we considered it likely that in macrophages the vesicular trafficking of HSV-1 might be extraordinary, and therefore restricts a productive infection. To directly visualize HSV-1 entry in macrophages and fibroblasts, we performed electron microscopy of macrophages and fibroblasts, which were infected with HSV-1 (MOI 250) for 30 min. We chose 30 min, as this is the time that HSV-1 requires to enter cells and first incoming capsids to reach the nuclear envelope of fibroblasts or epithelial cells^11,32–34^. Indeed, 30 min after infection we detected first viral capsids at the nuclear envelope of fibroblasts, but no newly produced viral capsids (Supplementary Fig. 2). Therefore we concluded that 30 min is an ideal time point to analyze the early internalization and intracellular distribution of HSV-1 within the cell. As expected, we found in macrophage and fibroblast cell cultures HSV-1 virions close to the plasma membrane (Fig. 2d). In macrophages, about 30% of all particles were found in endosomes and significantly more particles, about 50% of all particles, were found in multivesicular bodies (Fig. 2d, e). We could hardly detect viral capsids in the cytosol of macrophages (Fig. 2d, e). In fibroblasts, HSV-1 was detectable in vesicular compartments like early endosomes, late endosomes, MVBs and/or lysosomes (Fig. 2d). In addition electron microscopy revealed cytosolic capsids after 30 min of infection (Fig. 2d), indicating fusion of the viral envelopes with host membrane and successful capsid delivery into the cytosol. From these results we concluded that after HSV-1 is internalized by macrophages, it passes early and late endosomes and accumulates in MVBs but cannot deliver its capsid through the outer limiting membrane to the cytosol. This phenomenon might be responsible for limited productive infection in macrophages.

To determine why HSV-1 fails to release its capsid through the outer limiting MVB membrane in macrophages, we analyzed our microarray data for differences in membrane-modulating proteins. Strikingly we found overexpression of phospholipase C subtypes, neutral sphingomyelinase (Nsm, *Smpd3*) and acid ceramidase (aCDase, *Asah1*) in macrophages (Fig. 2f) in comparison to fibroblasts. Differential expression of these proteins was independent of IFN-I (Fig. 2f). Using real-time polymerase chain reaction (RT-PCR) and Western blot analysis we confirmed the high expression of *Asah1* mRNA and cleaved, i.e., activated, aCDase protein in macrophages compared to fibroblasts (Fig. 2g, h, Supplementary Fig. 7). From these experiments we concluded that macrophages accumulate HSV-1 in MVBs without being productively infected. This strong antiviral activity of macrophages correlated with aCDase expression.

### aCDase limits HSV-1 infection and prevents disease

**Fig. 3:**
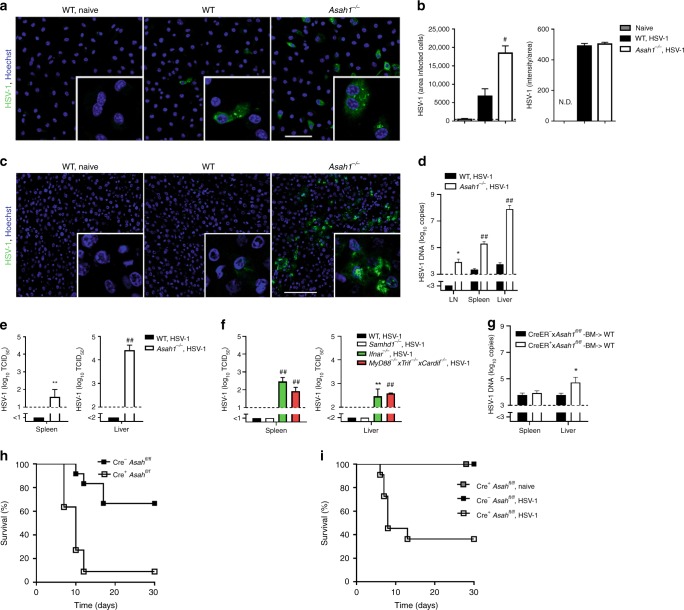
aCDase limits HSV-1 infection and prevents disease. **a**, **b** Representative images (**a**) and quantification (**b**) of immunofluorescence from wild-type (WT) and *Asah1*^−/−^ bone marrow derived macrophages (BMDMs) infected with HSV-1 for 6 h (multiplicity of infection [MOI] 10, *n* = 5, scale bar 50 µm, two-tailed student’s *t*-test). N.D.: not done. **c** Immunofluorescence of livers from WT and *Asah1*^−/−^ mice that were infected with 7 × 10^7^ plaque forming units (PFU) HSV-1 and analyzed after 12 h (*n* = 6–9, scale bar 100 µm). **d**, **e** Real-time polymerase chain reaction (RT-PCR) of lymph nodes (LN), spleens and livers (**d**, *n* = 7–10, 2-way ANOVA [Sidak’s multiple comparison]) and tissue culture infection dose 50 (TCID_50_, **e**, *n* = 4–6, two-tailed student’s *t*-test) of spleens and livers from WT mice and *Asah1*^−/−^ mice that were infected with 2 × 10^6^ TCID_50_ HSV-1 and analyzed on day 3. **f** TCID_50_ of spleens and livers from WT (*n* = 9), *Samhd1*^−/−^ (*n* = 4), *Ifnar*^−/−^ (*n* = 4), and *MyD88*^−/−^ *x Trif*^−/−^ *x Cardif*^−/−^ (*n* = 3, 2-way ANOVA [Tukey’s multiple comparison]) mice that were infected with 2 × 10^6^ TCID_50_ HSV-1 and analyzed on day 3. **g** RT-PCR of spleens and livers from bone marrow chimera mice receiving Cre^−^ *Asah1*^fl/fl^ or Cre^+^ *Asah1*^fl/fl^ bone marrow that were tamoxifen-treated, infected with 6 × 10^6^ TCID_50_ HSV-1 and analyzed on day 3 (*n* = 4–5, two-tailed student’s *t*-test). **h** Survival of tamoxifen-treated Cre^−^ *Asah1*^fl/fl^ and Cre^+^ *Asah1*^fl/fl^ mice which were infected intravaginally with 2 × 10^7^ TCID_50_ HSV-1 (*n* = 11–12, *p* = 0.0004, Log-rank [Mantel-Cox] test). **i** Survival of tamoxifen-treated Cre^***–***^ *Asah1*^fl/fl^ and Cre^+^ *Asah1*^fl/fl^ mice which were infected intravenously with 5 × 10^5^ TCID_50_ HSV-1 (*n* = 11–13, *p* = 0.0004, Log-rank [Mantel-Cox] test) or left uninfected (Cre^+^ *Asah1*^fl/fl^, naïve; *n* = 5). All data are shown as mean ± SEM. *p ≤ 0.05, **p ≤ 0.01, ^#^*p* ≤ 0.001, ^##^*p* ≤ 0.0001.

Since we identified that aCDase is a membrane modulating molecule that is highly expressed in macrophages, we next analyzed the antiviral activity of aCDase. WT macrophages showed limited propagation of HSV-1 (Fig. 3a, b). In strong contrast macrophages that lack aCDase (*Asah1*^−/−^*)* showed strong propagation of HSV-1 (Fig. 3a, b). Next we tested the role of aCDase in vivo. We infected WT and *Asah1*^−/−^ mice with HSV-1. In *Asah1*^−/−^ mice, HSV-1 showed overwhelming propagation after 12 h to 3 days (Fig. 3c, d) and infectious particles were produced in spleen and liver of *Asah1*^−/−^, but not control mice (Fig. 3e). Next we aimed to determine the importance of *Asah1* in relation to the known anti-HSV-1 effectors type I interferon (IFN-I)^35^ and SAMHD1^36^. Therefore we infected mice, which show limited production of IFN-I (*MyD88*^−/−^ *x Trif*^−/−^ *x Cardif*^−/−^ mice), limited response to IFN-I (*Ifnar*^−/−^ mice) and mice, which lack SAMHD1 (*Samhd1*^−/−^ mice), with HSV-1. Similarly to *Asah1*^−/−^ mice, *Ifnar*^−/−^ mice and *MyD88*^−/−^ *x Trif*^−/−^ *x Cardif*^−/−^ mice showed highly elevated HSV-1 DNA in lymph node, spleen and liver, when compared to control mice (Supplementary Fig. 3). In *Samhd1*^−/−^ mice HSV-1 DNA levels were elevated in the liver (Supplementary Fig. 3). In *Ifnar*^−/−^ mice and *MyD88*^−/−^ *x Trif*^−/−^ *x Cardif*^−/−^ mice, but not in *Samhd1*^−/−^ mice, we detected infectious HSV-1 particles in liver and spleen after HSV-1 infection, which was comparable to HSV-1 production in *Asah1*^−/−^ mice (Fig. 3f). From these data we considered aCDase to be an important antiviral effector mechanism.

To determine the macrophage-specific role of aCDase we generated bone marrow chimeras, in which *Asah1*^−/−^ bone marrow was transplanted into macrophage-depleted WT mice. In these mice macrophages repopulate from *Asah1*^−/−^ bone marrow. Chimeric mice with aCDase-deficient macrophages showed enhanced HSV-1 copies when compared to control mice (Fig. 3g), suggesting that indeed aCDase in macrophages contributed to control of HSV-1. To determine the overall importance of aCDase during systemic and local infection we infected Tamoxifen treated Cre-ER *Asah1*^***fl/fl***^ (inducible aCDase-deficient mice) with HSV-1. aCDase deficient Cre-ER *Asah1*^***fl/fl***^ mice died quickly after systemic and local intravaginal infection (Fig. 3h, i). Therefore we conclude that aCDase is an important effector mechanism against HSV-1, which acts in macrophages.

### Sphingosine protects against HSV-1 infection

**Fig. 4:**
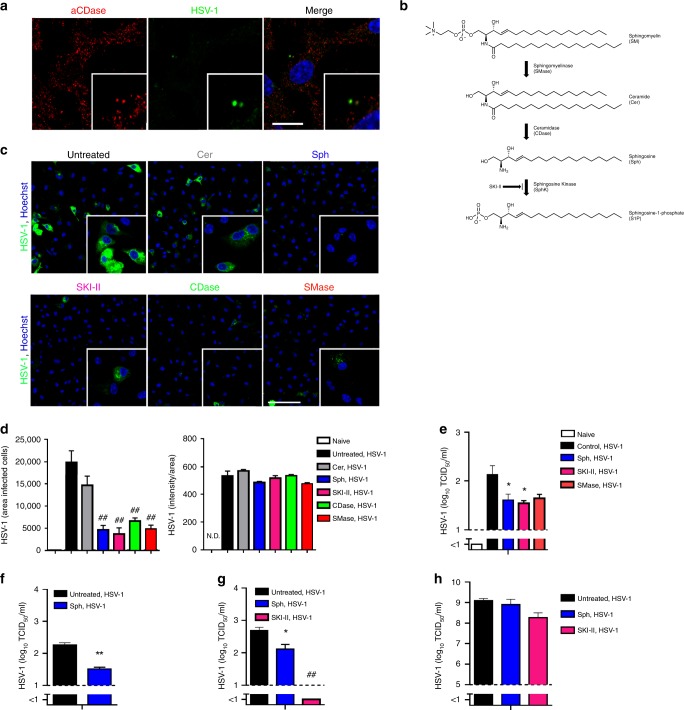
Sphingosine protects against HSV-1 infection. **a** Immunofluorescence of wild-type (WT) macrophages, which were infected with HSV-1 (multiplicity of infection [MOI] 10) and stained for acid ceramidase (aCDase) and HSV-1 (*n* = 5, blue represents Hoechst staining, scale bar 10 µm) after 30 min. **b** Schematic representation showing the metabolism of sphingomyelin (SM) to sphingosine-1-phosphate (S1P). **c**, **d** Representative image (**c**) and quantification (**d**) of immunofluorescence from bone marrow derived macrophages (BMDMs) that have been incubated for 30 min with β-D-galactosyl ceramide (Cer; 100 µM), D-erythro-sphingosine (Sph; 100 µM), sphingosine kinase inhibitor (SKI-II; 100 µM), ceramidase (CDase; 250 U/L) or sphingomyelinase (SMase; 6.5 U/ml) and subsequently infected with HSV-1 at a multiplicity of infection (MOI) of 100 (*n* = 5, scale bar 50 µm, one-way ANOVA [Dunnett’s multiple comparison]), fixed and stained after 6 h. N.D.: not done. **e** Tissue culture infection dose 50 (TCID_50_) of BMDMs that have been incubated for 30 min with D-erythro-sphingosine (Sph; 40 µM), sphingosine kinase inhibitor (SKI-II; 200 µM), or sphingomyelinase (SMase; 6.5 U/ml), subsequently infected with HSV-1 at a MOI of 1 and analyzed after 24 h (*n* = 3–4, one-tailed student’s *t*-test). **f** TCID_50_ of THP-1 cells that have been incubated for 30 min with D-erythro-sphingosine (Sph; 400 µM) and subsequently infected with HSV-1 at a MOI of 0.01, analyzed after 6 h (*n* = 3, two-tailed student’s *t*-test). **g** TCID_50_ of HeLa cells that have been incubated for 30 min with D-erythro-sphingosine (Sph; 100 µM), and sphingosine kinase inhibitor (SKI-II; 100 µM), and subsequently infected with HSV-1 at a MOI of 0.001 analyzed after 24 h (*n* = 3, one-way ANOVA [Dunnett’s multiple comparison]). **h** TCID_50_ of Vero cells that have been incubated for 30 min with D-erythro-sphingosine (Sph; 100 µM), and sphingosine kinase inhibitor (SKI-II; 100 µM), and subsequently infected with HSV-1 at a MOI of 0.01 analyzed after 24 h (*n* = 3, two-tailed student’s *t*-test). All data are shown as mean ± SEM. *p ≤ 0.05, **p ≤ 0.01, ^#^*p* ≤ 0.001, ^##^*p* ≤ 0.0001.

After analyzing aCDase deficiency, we investigated effects of increasing sphingolipid content during HSV-1 infection. From literature it is known that aCDase is usually active in lysosomes and MVBs^29–31,37^. To test whether aCDase directly interacts with HSV-1, we infected macrophages with HSV-1 and stained for aCDase and HSV-1 after 30 min. HSV-1 and aCDase were in close approximation, however we did not see a direct co-localization of aCDase with HSV-1 (Fig. 4a). This suggests that aCDase is not directly modulating HSV-1, but might modulate cellular components such as MVBs, which affect HSV-1 infection. Functionally, aCDase converts ceramide into sphingosine (Fig. 4b) and we considered that enhancement of sphingosine in MVBs was the reason for viral protection in WT macrophages against HSV-1. Indeed, addition of CDase 30 min before infection limits HSV-1 propagation, which suggests that the enzymatic function is most relevant for the antiviral activity (Fig. 4c, d).To reveal the role of sphingosine in HSV-1 infection, we treated macrophages with sphingosine (Sph), sphingomyelinase (SMase), and sphingosine kinase inhibitor (SKI-II), which are all involved in sphingolipid pathway and could enhance sphingosine levels^38^. Sphingosine treatment, which strongly elevated total sphingosine levels of primary macrophages and the Raw264.7 macrophage cell line (Supplementary Fig. 4), suppressed propagation of HSV-1 (Fig. 4c–e). Treatment with ceramidase, sphingomyelinase, and sphingosine kinase inhibitor resulted in smaller increases of cellular sphingosine levels and a corresponding suppression of HSV-1 propagation (Fig. 4c–e, Supplementary Fig. 4). From these data we concluded that enhanced amount of sphingosine was most likely the reason for antiviral activity in the presence of aCDase. To determine whether sphingosine acted cell type specific, we tested the antiviral activity of sphingosine in different cell types including human monocytes (THP-1), human cervix carcinoma cells (HeLa) and monkey kidney cells (Vero). To note, HSV-1 infects Vero cells via fusion at the plasma membrane while HeLa cells are infected after HSV-1 is endocytosed^10,11,39,40^. We found that sphingosine and/or SKI-II acted antiviral in undifferentiated THP-1 and HeLa cells (Fig. 4f, g). However we found limited antiviral activity of sphingosine in Vero cells (Fig. 4h), suggesting that the cell type and/or the route of infection might limit the antiviral activity of sphingosine. Taken together we concluded, that sphingosine, which is produced by aCDase in MVBs is antivirally active in macrophages.

### Sphingosine-rich intraluminal vesicles trap HSV-1

**Fig. 5:**
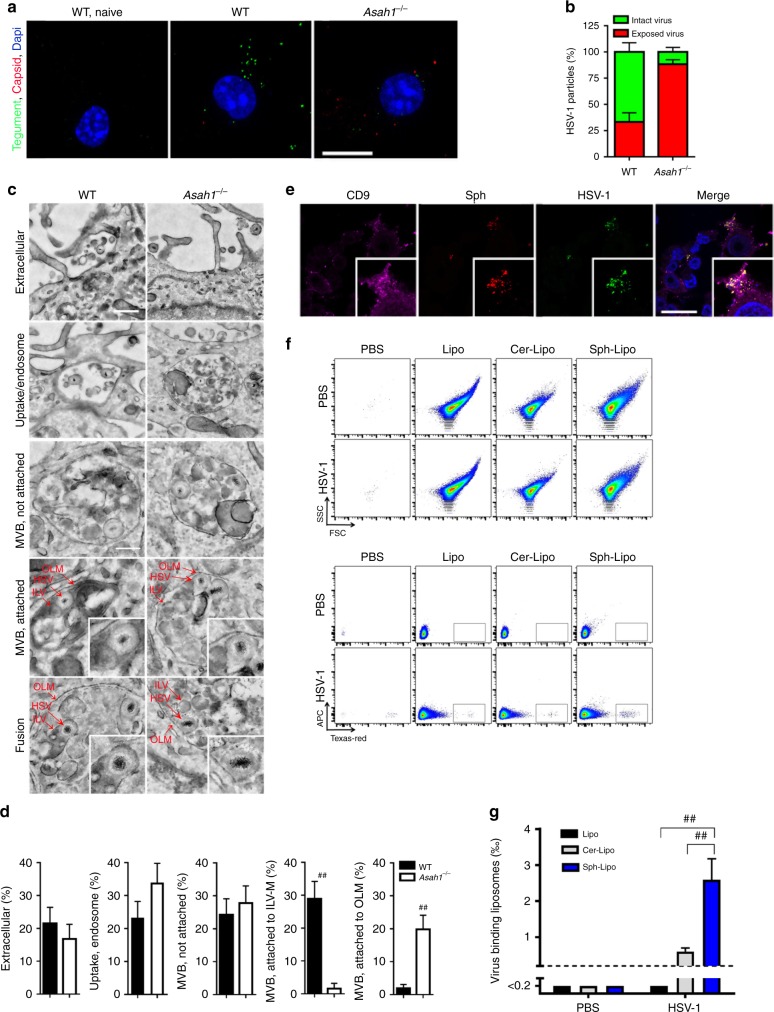
Sphingosine-rich intraluminal vesicles trap HSV-1. **a**, **b** Representative images (**a**) and quantification (**b**) of immunofluorescence from wild-type (WT) and *Asah1*^−/−^ bone marrow derived macrophages (BMDMs) incubated with HSV-1-VP16-GFP (multiplicity of infection [MOI] 30) for 30 min. Intact viral particles are visible through GFP-tagged tegument protein VP16 (green). Exposed capsids were stained with anti-VP5 (red, *n* = 17 images, one of two experiments is shown, scale bar 10 µm). **c**, **d** Representative electron microscopy images (**c**) and quantification (**d**) of WT and *Asah1*^−/−^ BMDMs infected with HSV-1 (MOI 250) analyzed after 30 min (*n* = 68 WT images and *n* = 63 *Asah1*^−/−^ images from three independent experiments; upper scale bar 400 nm, lower scale bar 200 nm, Mann-Whitney test). **e** Immunofluorescence of WT BMDMs, which were loaded with clickable *ω*-azido-sphingosine for 3 h, infected with HSV-1 (MOI 10) for 30 min and then stained for sphingosine, the intraluminal vesicle (ILV) marker CD9, HSV-1 anti-capsid and Hoechst (blue, *n* = 5, scale bar 20 µm). **f**, **g** Representative dot plots (**f**) and quantification (**g**) of control liposomes, ceramide-loaded liposomes and sphingosine-loaded liposomes that were incubated with fluorescently labeled HSV-1 for 10 min and then analyzed for HSV-1 binding in flow cytometry (*n* = 6, 2-way ANOVA [Tukey’s multiple comparison]). All data are shown as mean ± SEM. *p ≤ 0.05, **p ≤ 0.01, ^#^*p* ≤ 0.001, ^##^*p* ≤ 0.0001.

Next we aimed to determine the mechanism, how aCDase and sphingosine limit HSV-1 infection in macrophages. To this end, we incubated cells for 30 min with an HSV-1 carrying a fluorescently labeled tegument (HSV-1-VP16-GFP), and stained with an antibody for viral capsids (VP5^41^), which recognizes the uncoated capsid after virus membrane fusion. In this experimental setup capsid is only visible after dissociation of the tegument^11^. We mainly observed HSV-1-VP16-GFP puncta, in WT macrophages (Fig. 5a, b), indicating that the HSV-1 capsids were not released from the envelope. In strong contrast, in aCDase lacking (*Asah1*^−/−^*)* macrophages we detected almost exclusively exposed capsids without tegument (Fig. 5a, b), suggesting that the capsids were released from the envelope and the tegument due to virus infection. To get more insights into the reason for limited infection in WT macrophages, we performed electron microscopy experiments in WT and *Asah1*^−/−^ macrophages. WT and *Asah1*^−/−^ macrophages showed similar amounts of HSV-1 particles attached to the plasma membrane (Fig. 5c, d). In line, similar amounts of HSV-1 particles were detected in WT and *Asah1*^−/−^ macrophages in endosomes and MVB (Fig. 5c, d). In WT macrophages HSV-1 particles were located in close proximity to the ILVs, and in some cases appeared to fuse with ILVs (Fig. 5c, d). This correlated with trapping of HSV-1 in ILVs (Fig. 5c, d). In contrast, in *Asah1*^−/−^ macrophages, the HSV-1 envelopes were more frequently associated with the outer limiting membranes (OLMs) of the MVBs (Fig. 5c, d), so that membrane fusion here led to a productive release of capsids into the cytosol and infection of the cell. Therefore, we concluded that aCDase-derived sphingosine mediates binding of HSV-1 envelope to ILVs membrane, which results in trapping of the virus.

To prove that indeed sphingosine on ILVs interacted with HSV-1 we loaded WT macrophages with *ω*-azido-sphingosine, which can be visualized by a click reaction^42^. 3 h after sphingosine loading, we infected cells with HSV-1 and co-stained cells with the ILV marker CD9. Indeed, sphingosine accumulated in ILVs (Fig. 5e). As expected HSV-1 co-stained with these sphingosine-rich ILVs (Fig. 5e). To determine that indeed sphingosine is responsible for interaction between HSV-1 and ILVs, we generated liposomes and loaded them either with ceramide or sphingosine. Liposomes were then incubated with HSV-1 and binding of virions was analyzed after 10 min by flow cytometry. Control and ceramide-liposomes did not bind HSV-1 (Fig. 5f, g). In contrast, sphingosine-liposomes bound HSV-1 (Fig. 5f, g). Concluding, we found that the antiviral effects of aCDase are mediated via fusion of viral envelop to sphingosine containing ILVs of MVBs.

### IRF8, but not IFN-I signaling, induces aCDase expression

**Fig. 6:**
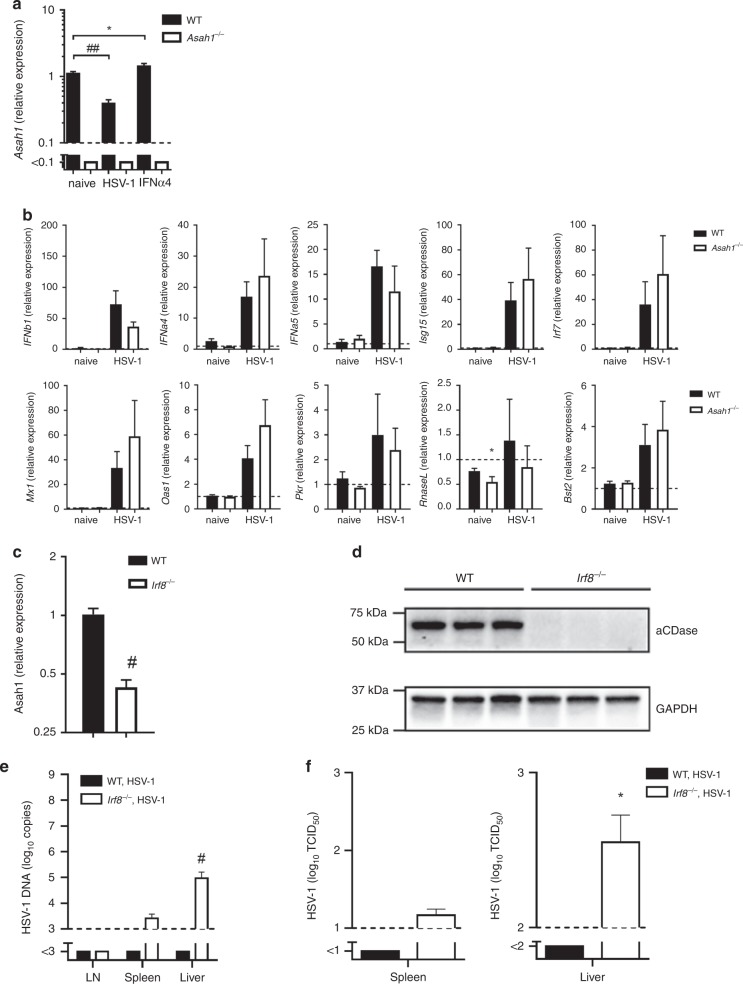
IRF8, but not IFN-I signaling, induces aCDase expression. **a** Real-time polymerase chain reaction (RT-PCR) of wild-type (WT) and *Asah1*^−/−^ bone marrow derived macrophages (BMDMs) that were left untreated, infected with herpes simplex virus type 1 (HSV-1) at a multiplicity of infection (MOI) of 1 for 24 h or infected and treated with 100 units interferon-α4 (IFN-α4; *n* = 10, one-way ANOVA [Dunnett’s multiple comparison]). **b** RT-PCR of WT and *Asah1*^−/−^ BMDMs that were left untreated or infected with HSV-1 at an MOI of 1 for 24 h (*n* = 10, two-tailed student’s *t*-test). **c**, **d** RT-PCR for *Asah1* (**c**, *n* = 5, two-tailed student’s *t*-test) and Western blot for aCDase (**d**, *n* = 3) of spleen tissues from WT and interferon regulatory factor 8 deficient (*Irf8*^−/−^) mice that were left untreated. **e**, **f** RT-PCR (**e**) of lymph nodes (LN), spleens and livers and tissue culture infection dose 50 (TCID_50_, **f**) of spleens and livers from WT and *Irf8*^−/−^ mice that were infected with 2 × 10^6^ TCID_50_ HSV-1 and analyzed on day 3 (*n* = 5-6, two-tailed student’s *t*-test). All data are shown as mean ± SEM. *p ≤ 0.05, **p ≤ 0.01, ^#^*p* ≤ 0.001, ^##^*p* ≤ 0.0001.

Our data show that the *Asah1* gene encoding aCDase is a crucial factor involved in antiviral protection against HSV-1. Next, we aimed to define, how aCDase expression is induced. We aimed to analyze whether *Asah1* is also induced by IFN-I. The results show that *Asah1* expression was hardly influenced by IFN-I, nor did *Asah1* influence the induction of IFN-I or interferon induced genes (Fig. 6a, b). Therefore we concluded that *Asah1* is not specifically upregulated during infection, but shows already basal activity in macrophages before infection. To get insights how *Asah1* expression is upregulated in macrophages we considered that interferon regulatory factor 8 (IRF8) might play a role in this process. IRF8 is a factor, which induces natural resistance in macrophages during their development^43,44^. Furthermore, it was shown that IRF8 regulates the expression of aCDase during the induction of apoptosis in cancer cells^45^. RT-PCR analysis of myeloid pre-cursor cells in the bone marrow revealed IRF8-dependent upregulation of *Asah1* (Supplementary Fig. 5). To determine the role of IRF8 in regulating *Asah1* in mature macrophages, we analyzed *Asah1* expression in the spleen, an organ showing high numbers of macrophages. RT-PCR and Western blot analysis revealed limited levels of *Asah1* mRNA and aCDase precursor protein in spleens of *Irf8*^−/−^ mice when compared to WT spleens (Fig. 6c, d, Supplementary Fig. 8). In line, *Irf8*^−/−^ mice showed elevated levels of HSV-1 DNA as well as increased production of viral particles after infection with HSV-1 (Fig. 6e, f). It has previously been reported, that IRF8 expression in human blood monocytes decreases with age, which correlates with decreased expression of interferon stimulated genes and might be a reason for impaired antiviral resistance to influenza A virus (IAV)^46^. Whether this reduced IRF8 expression influences also aCDase dependent virus control remains to be studied. We concluded that *Asah1* is not induced during infection or by IFN-I, however IRF8 regulates *Asah1* expression during development of macrophages.

## Discussion

Macrophages are one of the most important effector cells of the innate immune system^5,47^. During infection with viruses such as vesicular stomatitis virus (VSV), lymphocytic choriomeningitis virus (LCMV), adenovirus or mouse hepatitis virus, macrophages play a major protective role in limiting infection and preventing severe disease^2–4,48–50^. In particular, tissue resident macrophages such as Kupffer cells in the liver clear a range of systemic virus infections^4,48^. Molecular mechanisms explaining this strong antiviral activity are presently unknown. Here we found that macrophages are essential for the control of HSV-1. With our in vitro data, we linked the strong antiviral activity of macrophages to the expression of aCDase. aCDase produced sphingosine-rich ILVs in MVBs. Once HSV-1 is taken up into MVBs, sphingosine-rich ILVs bind to HSV-1 particles and thereby trap them in MVBs and prevent infection of the cell. Expression of aCDase was not regulated by IFN-I, but was induced in macrophages in response to IRF8 signaling. While we focused in our manuscript on the role of aCDase in macrophages, our in vivo data suggest that aCDase exerts antiviral activity probably also in other myeloid or even none-myeloid cell types.

It remains to be explored whether the restriction mechanism of sphingosine-rich ILVs is specific for HSV-1 or whether also other viruses are sensitive to sphingosine-rich ILVs. Interestingly, VSV, the flaviviruses Japanese encephalitis virus (JEV) and yellow fever virus (YFV) fuse with ILVs in MVBs^51,52^. Le Blanc et al.^51^ and Nour et al.^52^ considered the release of viral capsids into ILVs a relevant step in virus life cycle, and suggested that back-fusion of ILVs, harboring capsids, with the OLMs of MVBs leads to productive infection. Our data show that although back-fusion might occur in macrophages, fusion of viral membrane with ILV membrane is rather a cellular mechanism to limit infection of the cell. Further experiments are required to determine whether other enveloped viruses and non-enveloped viruses are also restricted by sphingosine in MVBs.

Viruses frequently have an acidic isoelectric point at low pH^53^, implying that these viruses are negatively charged at physiological pH. The isoelectric point of HSV-1 is around 4.9^54^. In contrast, sphingosine is net positively charged at neutral or acidic pH, as occurring in MVBs. We suggest that sphingosine gives ILVs a positive charge, which facilitates the interaction with negatively charged HSV-1. Indeed it was shown that sphingosine containing vesicles interact with negatively charged membranes at the plasma membrane or in late endosomes and accelerates fusion of these membranes^55,56^. More biophysical work is required to better understand the interaction of HSV-1 with sphingosine.

We compared the in vivo phenotype of *Asah1*^−/−^ mice during HSV-1 infection to several other mouse strains, which show limited production of IFN-I (*MyD88*^−/−^ *x Trif*^−/−^ *x Cardif*^−/−^ mice), limited response to IFN-I (*Ifnar*^−/−^ mice) or lack SAMHD1 (*Samhd1*^−/−^ mice). SAMHD1 was previously reported to be of importance for HSV-1 control in myeloid cells^36,57^ and anti-viral effects can be evaded by some herpesviruses^58^. Viral replication in *Asah1*^−/−^ was comparable to *MyD88*^−/−^ *x Trif*^−/−^ *x Cardif*^−/−^ mice and *Ifnar*^−/−^ mice and even enhanced to that in *Samhd1*^−/−^ mice. These findings indicate that *Asah1* is an important and major innate effector mechanism. We observed increased HSV-1 DNA in livers of *Samhd1*^−/−^ mice. This is in line with other groups which determined an important role of SAMHD1 on HSV-1 control in vitro^36,57,58^. How this lack of HSV-1 control influences disease onset after HSV-1 infection needs to be determined in the future. While SAMHD1 and IFN signaling counteract mainly viral replication and production of infectious particles, we think that aCDase protects macrophages from infection with HSV-1 immediately after the virus is taken up.

Our electron microscopy studies indicate that HSV-1 fuses with ILVs. We consider that trapped viral particles are unable to leave MVBs and are targeted to lysosomes for degradation. This pathway of degradation might also facilitate antigen-presentation and immune activation, as high numbers of tetraspanins and MHC-II molecules are contained within ILVs^23,59^. More studies are required to identify the relationship of aCDase, sphingosine and antigen-presentation on MHC-II. In our study we focused mainly on the role of sphingosine and aCDase in macrophages. In addition, aCDase is expressed in several other tissues, especially in lung, colon and stomach as well as spleen^60^ (Supplementary Fig. 6). Cells in these organs are involved in building a barrier, which protects against invading viruses. We consider it likely that in addition to macrophages, potentially also other cell types express aCDase in MVBs and are thereby protected against invading viruses.

From our data we suggest enhanced virus infections in patients with Farber disease, which are deficient in activity of aCDase^61^. While such associations were not determined yet, patients with Farber disease have a limited life span and die early after birth^61^. Akin to other innate or adaptive deficiencies, a limited lifespan of the patients may obscure a link between innate or adaptive deficiencies to infectious agents^62^. Whether other mutations, which affect the activity of aCDase, are widespread in the population and whether such mutations influence the susceptibility to viral infections will be subject to future studies.

In conclusion, we found that macrophages are essential to survive an infection with HSV-1. Mechanistically, macrophages took up HSV-1 and accumulated it in MVBs. aCDase in macrophages enriched ILVs with sphingosine. Once HSV-1 entered MVBs sphingosine-rich ILVs trapped HSV-1 and prevented productive infection of the cells.

## Methods

### Mice

For acid ceramidase 1 (*Asah1*) deficient heterozygous mice, the exon-intron organization of the gene was established on the basis of the *Asah1* cDNA sequence. A targeting vector was constructed, including *Asah1* genomic sequences on the C57BL/6 genetic background, a long homology region of 5.8 kb and a short homology region of 1.7 kb. It also included two loxP sites flanking *Asah1* exon 1, a neomycin gene flanked by flipase recognition target sites for positive selection of homologous recombination, and a diphtheria toxin, a negative-selection marker to detect insertions and nonhomologous recombinant embryonic stem cell clones. This targeting vector was inserted into C57BL/6 embryonic stem cells by homologous recombination. Offspring were crossed with CreER mice (Gt[ROSA]26Sor^tm9[cre/ESR1]Arte^; Taconic Artemis Pharmaceuticals [Köln, Germany]). In some experiments we used mice in which acid ceramidase deficiency could be induced (Cre × *Asah1*^*fl/fl*^). CreER x *Asah1*^*fl/fl*^ offspring express a Cre recombinase under the tamoxifen promoter and the *Asah1* gene with two LoxP sites flanking exon 1. Administering tamoxifen thus led to the expression of Cre recombinase and loss of function mutation by deletion of exon 1 in the *Asah1* gene. In this model, knock out of *Asah1* occurs right before infection. Potential developmental defects due to long term knock out of *Asah1* are excluded in this model.

*Asah1*^*fl/fl*^ x EIIa-Cre mice carry a Cre transgene under the control of the adenovirus EIIa promoter that targets expression of Cre recombinase to the early mouse embryo, and are used for germ line deletion of *Asah1* gene, referred to as *Asah1*^−/−^ mice. *Asah1*^−/−^ mice were on C57BL/6J background. Sibling animals with the genotype *Asah1*^***+/+***^ were used as control animals. We bred *Irf8*^−/−^, *Ifnar*^*−/−*^, *MyD88*^*−/−*^ *x Trif*^*−/−*^ *x Cardif*^*−/−*^ and *Samhd1*^−/−^ mice on C57BL/6J background. *Samhd1*^−/−^ mice were originally developed by Prof. Jan Rehwinkel (MRC Human Immunology Unit, MRC Weatherall Institute of Molecular Medicine, University of Oxford, Oxford, England)^63^.

All animals were housed in single ventilated cages. Animal experiments were authorized by the Landesamt für Natur, Umwelt und Verbraucherschutz (LANUV) Nordrhein-Westfalen and in accordance with the German law for animal protection and/or according to institutional guidelines at the Ontario Cancer Institute of the University Health Network.

### Cell lines, bone marrow macrophages

Raw264.7 cells were purchased from the American Type Culture Collection (ATCC, Manassas, VA, USA, #: ATCC: TIB-71). THP-1 cells were purchased from the German Collection of Microorganisms and Cell Cultures (DSMZ, Braunschweig, Germany, #: DSMZ: ACC 16). Vero cells were provided by the Ontario Cancer Institute (Toronto, ON, Canada). HeLa cells were provided in house (University Clinic Essen, Essen, Germany). For the generation of macrophages, bone marrow was isolated from mice and cultured for 9 days with macrophage colony-stimulating factor (M-CSF). Cells were plated on new cell culture plates and the experiment was started the next day. For generation of primary fibroblasts, lungs were isolated from mice and digested using DNase/Liberase. Fibroblasts were harvested after 6 days of culture and experiment was started the subsequent day.

### Viruses

We used HSV-1(17+)Lox and HSV-1(17+)Lox-CheVP26 in which monomeric Cherry has been fused to the N-terminus of the small capsid protein VP26^64^, HSV-1F (kindly provided by Prof. Hartmut Hengel, Institute of Virology, Freiburg, Germany) and HSV-VP16-GFP^65^ (kindly provided by Dr. Yohei Yamauchi, University of Bristol, United Kingdom).

### Reagents

D-erythro-sphingosine (C18, 860490) and D-Galactosyl-β1-1’-N-Nervonoyl-D-erythro-sphingosine (C24:1, β-D-Galactosyl Ceramide, 110759), were obtained from Avanti Polar Lipids (Alabaster, AL, USA). Glycoceramidase (E9030-100MUN), sphingomyelinase (S8633-25UN), Cy3-linked DBCO (777366-1 mg) and DAPI (D9542) were purchased from Millipore Sigma (Darmstadt, Germany). Recombinant mouse Asah2 (3558-AH) was purchased from R&D Systems (Minneapolis, MN, USA). The sphingosine kinase inhibitor SKI-II (CAS # 312636-16-1; Cat # 2097) was obtained from Tocris Bioscience (Bristol, United Kingdom) and tamoxifen (CAS # 10540-29-1, Cat # T5648-5G) and cornoil (CAS # 8001-30-7, Cat # C8267-500ML) from Millipore Sigma. Clodronate and control liposomes were purchased from Liposoma (CP-005-005, Amsterdam, Netherlands). Clickable *ω*-azido-sphingosine ((2*S*,3*R*,*E*)-2-amino-18-azidooctadec-4-ene-1,3-diol) for click chemistry^42^ staining was synthesized in-house by Julian Fink.

### Tamoxifen treatment

Tamoxifen was dissolved in corn oil. Eight, six, and four days before an experiment, Cre^-^ × *Asah1*^*fl/fl*^ control animals and Cre^+^ × *Asah1*^*fl/fl*^ animals were treated with 4 mg tamoxifen (in 100 μL corn oil) intraperitoneally.

### Depletion of macrophages

For macrophage-depleted mouse experiments, mice were treated intravenously with 50 mg/kg clodronate to deplete tissue resident macrophages. After 3 days, mice were ready to be used in experiments.

### Survival experiments

To analyze the effect of macrophage/ sphingosine deficiency on the course of infection, we performed survival experiments. For this purpose, animals were infected with a dose that was sublethal for WT animals. Animals were checked daily, killed with corresponding termination criteria, and counted as dead. Termination criteria were body weight, general condition, spontaneous behavior and clinical findings.

### Bone marrow chimera mice

For bone marrow chimera experiments, 10–12 weeks old female C57BL/6J mice were irradiated with 9.5 Gy. On the following day, bone marrow from donor mice was isolated under sterile conditions and i.v. administered. On day 10 after irradiation, mice were treated with clodronate liposomes to deplete tissue resident macrophages. After 50 days of reconstitution mice were ready to be used in experiments.

### Infection of cells and animals

For in vitro infection, the virus was added to the cell cultures. After incubation for 1 h, the supernatant was washed away and new medium was added. The supernatant was collected at various times after infection. For intravenous infection, 100 µL of the virus was injected into the tail vein. For intravaginal infection, 20 µL of the virus was pipetted into the vagina of anesthetized mice. The vagina was briefly closed with skin glue to reduce variability of inoculation time and volume.

### Immunofluorescence microscopy

Immunofluorescence was used to detect viruses in cells and organs. For in vitro experiments, cells were seeded in 24-well plates, each containing a coverslip. After 24 h, the cells were correspondingly infected, fixed after incubation, and labeled with antibodies. Organs of infected animals were snap-frozen, and 8 μm thick sections were recovered on a microscope slide. The sections were fixed, and virus was visualized with virus-specific antibodies. The following primary antibodies were used: anti-HSV-1 capsid (SY4563^34^, anti-capsid, 1:100), anti-HSV-1 glycoprotein (hu2c^66^, anti-glycoprotein gB, 1:200) or anti-HSV-1 VP5 (NC-1^41^, anti-capsid, 1:500). Alternatively, virus was directly labeled with a dye (e.g., Cy3; carboxylfluorescein succinimidyl ester [CFSE]) was used. Zeiss ELYRA PS.1 SIM/PAL-M/STORM/TIRF and LSM710 (ZEISS, Oberkochen, Germany), was used to acquire images.

### Immunofluorescence microscopy of sphingosine

Immunofluorescence was used to detect sphingosine in cells. Cells were seeded in 24-well plates, each containing a coverslip. After 24 h, the cells were correspondingly treated, fixed after incubation, and labeled with antibodies. As primary antibody monoclonal anti-sphingosine antibody (clone NHSPH, ALF-274042010, Alfresa Pharma Corporation, Osaka, Japan, 1:1000) and as secondary antibody Cy3-coupled anti-IgM F(ab)2 fragments (Jackson ImmunoResearch, Ely, United Kingdom, 1:500) were used. Samples were examined with a Leica TCS-SP5 confocal microscope equipped with a 100× lens, and images were analyzed with Leica LCS software version 2.61 (Leica Microsystems, Mannheim, Germany). Fluorescence intensity was determined in 100 randomly selected cells/sample (total of 300 cells). We determined the fluorescence of the cell periphery.

### Detection of HSV in cell supernatant/organs by TCID_50_ assay

For tissue culture infection dose 50 (TCID_50_) Vero cells were seeded onto a 96-well plate and let grow to confluence overnight. To determine the viral titer, cell culture supernatant or organ suspension was titrated and transferred to the 96-well plate. After 7 days of incubation, the plaque positive wells were counted, and the number of infectious particles per ml of supernatant or organ was determined.

### Detection of HSV in organs by qRT-PCR

For quantitative real-time polymerase chain reaction (qRT-PCR) DNA was isolated from organs using the innuPREP Virus DNA Kit (Order #: 845-KS-4600050, Analytik Jena, Jena, Germany). RealStar HSV PCR Kit 1.0 CE (Order #: 061013, Altona Diagnostics, Hamburg, Germany) was used to quantify the amount of HSV-1 genomes per organ. A LightCycler 480 Instrument (Roche Life Science, Basel, Switzerland) was used for detection.

### Capsid uncoating assay of herpes simplex virus

Cells were incubated with HSV-1 expressing GFP at the tegument protein VP16 (HSV-1-VP16-GFP^65^). After 30 min of incubation, cells were fixed and stained for HSV capsid with anti-VP5 (NC-1^41^, 1:500) and DAPI. Images were acquired on Leica SP8 microscope (Wetzlar, Germany) and analyzed using CellProfiler by segmentation and counting of single VP16-GFP or VP5 puncta.

### Sphingosine-containing liposomes for FACS analysis

Liposomes were prepared by sonication as previously described^67^. Phosphatidylcholine (PC), cholesterol (Ch), sphingomyelin (SM), ceramide (Cer) and D-sphingosine (Sph) were from Millipore Sigma. The lipid composition of the liposomes was as follows: control-liposomes (PC: Ch: SM 33.3:33.3:33.3 mol/%), ceramide-rich liposomes (PC: Ch: SM: Cer 33:33:24:10 mol/%) and sphingosine-rich liposomes (PC: Ch: SM: Sph 33:33:24:10 mol/%).

### Isolation of total RNA, reverse transcription and qRT-PCR

Total RNA was isolated from cells using Trizol. Complementary DNA synthesis was performed with the QuantiTect Reverse Transcription Kit *(*Qiagen, Hilden, Germany).

For quantitative real-time polymerase chain reaction (qRT-PCR) either Fast SYBR Green Master Mix (Applied Biosystems, Darmstadt, Germany) or TaqMan Fast Universal PCR Master Mix (ThermoFisher Scientific) was used on the 7500 Fast Real-Time PCR System (Applied Biosystems). For analysis, the expression levels of all target genes were normalized against glyceraldehyde 3-phosphate dehydrogenase (Gapdh; ΔCt). Relative gene expression values were calculated with the ΔΔCt method. Following primers were used: *Gapdh* (Mm99999915_g1, ThermoFisher Scientific), *Gapdh* (QT01658692, Qiagen), *Asah1* (Mm00480021_m1, ThermoFisher Scientific) IRF7 (Mm00516788_m1, ThermoFisher Scientific), Isg15 (QT00322749, Qiagen), RnaseL (QT01066086, Qiagen), Tetherin/Bst2 (QT01066184,Qiagen), IFNα4 (QT01774353, Qiagen), IFNα5 (QT00327656, Qiagen), IFNβ1 (QT00249662, Qiagen), Mx-1 (QT01064231, Qiagen), Oas1 (QT01056048, Qiagen) and PKR (QT00162715, Qiagen).

### Click chemistry

Clickable *ω*-azido-sphingosine ((2*S*,3*R*,*E*)-2-amino-18-azidooctadec-4-ene-1,3-diol) was used for click chemistry reaction as described previously^42,68^. Briefly, cells were seeded and grown to confluence. *ω*-azido-sphingosine was added to cell culture medium (100 μmol/L) for 3 h. Cells were washed with PBS and then Cy3-linked DBCO (8 μg/ml diluted in PBS) was added for 7 min at room temperature. Samples were washed 3x with PBS and medium was added. Cell were further analyzed as described.

For detailed synthesis and analysis of *ω*-azido-sphingosine see methodical information “chemical synthesis of *ω*-azido-sphingosine” in Supplementary Information and Supplementary Figs. 9–20.

### Microarray

Total RNA preparations were checked for RNA integrity with an Agilent 2100 Bioanalyzer. All samples in this study exhibited high-quality RNA Integrity Numbers (RIN 9.8-10). Synthesis of cDNA and subsequent fluorescence labeling of cRNA were performed according to the manufacturer’s protocols (One-Color Microarray-Based Gene Expression Analysis/Low Input Quick Amp Labeling; Agilent Technologies, Waldbronn, Germany). Briefly, 100 ng of total RNA was converted to cDNA, followed by in vitro transcription and incorporation of Cy3-CTP into nascent cRNA. After fragmentation, labeled cRNA was hybridized to AgilentSurePrint G3 Mouse GE 8×60k microarrays for 17 h at 65 °C and scanned as described in the manufacturer’s protocol. Signal intensities on 20-bit TIFF images were calculated by Feature Extraction software (FE, Vers. 10.7.1.1; Agilent Technologies). Data analyses were conducted with GeneSpring GX software (Vers. 10.5; Agilent Technologies). Probe signal intensities were quantile-normalized across all samples for reduction of inter-array variability. Input data preprocessing was concluded by baseline transformation to the median of all samples.

To ensure innate immune activation macrophages and fibroblasts were infected with LCMV and treated with IFN-α4. To analyze the microarray data a gene set enrichment analysis with GSEA (Broad Institute, v3.0^69^) using the Gene Ontology Cellular Components (CC) gene set database (c5.cc.v6.2^70^, accessed on 15.07.19) was performed using default settings. Results were further subjected to network analysis using the Enrichment Map plugin v3.2.0 in Cytoscape v3.7.1 with the following settings: FDR q-value cutoff 0.05, p-value cutoff 1, Metric: Jaccard+Overlap Combined (50/50) with cutoff 0.375. Clusters were identified by the AutoAnnotate plugin v1.3 and labeled manually for visualization. For visualization gene sets upregulated in LCMV-infected and interferon-treated macrophages or fibroblasts were sorted to the left or right side of the diagram respectively. Several membrane modulating proteins from the families of sphingolipids (including gangliosides), cholesterol and phosphatidylcholine were considered for simplified comparison of macrophages and fibroblasts (see Supplementary Table 2).

### Virus binding to liposomes

Liposomes were incubated with fluorescently labeled virus. Samples were diluted and further analyzed in flow cytometry (LSR Fortessa). Liposomes were detected by size and granularity. Frequency of liposomes, which bound the virus was determined by fluorescence according to the gating shown in Fig. 5f. Data was analyzed using the software FlowJo (FlowJo LLC).

### FACS Sorting

Bone marrow was isolated from tibia and femur of mice and erythrocytes were lysed. To sort pre-progenitor cells staining for linage markers anti-CD3e (Cat # 45-0031-80, eBioscience, 1:50), anti-GR1 (Cat # 108428, BioLegend, 1:400), anti-Mac-1 (Cat # 101228, BioLegend, 1:400), anti-CD4 (Cat # 101228, BioLegend, 1:200), anti-CD8a (Cat # 100734, BioLegend, 1:100), anti-TCRb (Cat # 109228, BioLegend, 1:100), anti-NK1.1 (Cat # 45-5941-82, eBioscience, 1:100), anti-B220 (Cat # 103236, BioLegend, 1:100) and anti-CD19 (Cat # 115534, BioLegend, 1:200) was performed. Additionally anti-CD16/32 (Cat # 101305, BioLegend, 1:200), anti-CD41 (Cat # 133906, BioLegend, 1:100), anti-Sca-1 (Cat # 108114, BioLegend, 1:200), anti-c-kit (Cat # 47-1171-82, Invitrogen, 1:50), anti-CD105 (Cat # 120412, BioLegend, 1:50) and anti-CD150 (Cat # 115910, BioLegend, 1:400) were stained to further classify cells. To sort immature granulocytes, mature granulocytes and monocytes anti-Ly6G/Ly6C (Cat # 17-5931-82, Invitrogen, 1:100), anti-CD11b (Cat # 25-0112-82, eBioscience, 1:100) and anti-CD115 (Cat # 11-1031-85, eBioscience, 1:100) were used. Gating strategy was performed as shown in Supplementary Fig 5.

### Western blot for aCDase protein expression

Frozen mouse spleen or cultured primary mouse cells were lysed in radioimmunoprecipitation buffer containing 1x HALT Protease Inhibitor Cocktail and 50 mM EDTA (Thermo Fisher, Cat. no. 78430) using 100 µl lysis buffer per 10 mg organ or 30 µl lysis buffer per 10^6^ cells respectively. Spleens were homogenized using a homogenizer (Qiagen Tissue Lyser II, Cat. no. 85300) for 2 min at 20 Hz. Lysates were then centrifuged for 20 min at 20,000 x g at 4 °C, keeping the supernatants. Protein concentration was measured using detergent compatible (DC) Protein Assay (Bio-Rad, Cat. no. 5000112), standardized to 4 µg/µl and prepared for SDS-PAGE by addition of 4x Laemmli sample buffer (Bio-Rad, Cat. no. 1610747), incubation at 95 °C for 5 min followed by cool-down on ice. 30 µg per organ or 10 µg per cell lysate was loaded per lane into a precast TGX AnyKD Stain-free gel (Bio-Rad, Cat. no. 4568126), using Bio-Rad AllBlue protein standard (Cat. no. 1610373) and ran for 45 min at 140 V in Tris/glycine/SDS running buffer (Bio-Rad, Cat. no. 1610732). Transfer was performed using the Trans-Blot Turbo system (Bio-Rad, Cat. no. 1704150) with Mini PVDF Transfer Packs (Bio-Rad, Cat. no. 1704156). Membranes were blocked in Pierce StartingBlock (TBS) Blocking Buffer (Thermo Fisher, Cat. no. 37542) for 2 h and primary antibody was incubated shaking at 4 °C for 2 days: 1:500 for anti-aCDase rabbit polyclonal (Proteintech, Cat. no. 11274-1-AP) for spleens, or 1:500 anti-aCDase rabbit polyclonal (ProSci, Cat. no. 4741) for cell lysates, and 1:1000 for anti-GAPDH mouse (Meridian Life Science, Cat. no. H86504M) diluted in blocking buffer. Membranes were washed three times with Tris-buffered saline-0.1 % Tween 20 (TBS-T) and incubated with secondary antibody at 1:5000 (from GE Life Sciences, anti-Rabbit IgG, HRP-linked, Cat. no. NA934 or anti-mouse IgG, HRP linked, Cat no. NA931) diluted in 25 % blocking buffer in TBS-T shaking at room temperature for 2 h. After washing twice in TBS-T and twice in TBS membranes were incubated with Pierce ECL (Thermo Fisher, Cat. no. 32106) for 5 min and imaged on a Bio-Rad ChemiDoc MP (Cat. no. 1708280) with ImageLab version 6.0.1 (Bio-Rad).

### Histology

Histological analysis of snap-frozen liver and spleen tissues or cell cultures was performed with monoclonal fluorescence labeled antibodies against HSV-1-capsid (SY4563, 1:100) anti-HSV-1 (hu2c^66^, anti-gB, 1:200), anti-F4/80 (12-4801-82, eBioscience, San Diego, CA, USA, 1:100), anti-Asah1 (4741, ProSci, San Diego, CA, USA, 1:200), anti-CD9 (124809, Biolegend, San Diego, CA, USA, 1:100) and anti-sphingosine (clone NHSPH, ALF-274042010, Alfresa Pharma Corporation, Osaka, Japan, 1:1000). Nuclei were stained using Hoechst 33342 (Cat # B2261-100MG, Sigma, 1:5000). Zeiss ELYRA PS.1 SIM/PAL-M/STORM/TIRF and LSM710, Leica SP8 gSTED and FLIM, Leica TCS-SP5, or Keyence BZ-9000 (Keyence, Osaka, Japan) were used to acquire images. Quantification of histology was done using standard procedures in Fiji software version 1.52e (Image J, NIH, Bethesda, MD, USA) or Cell Profiler^71^. In Fiji we used the raw images, applied a mean filter of two/ten to exclude noise. We took the total signal area and the intensity per area after an empirically fixed threshold for further evaluation.

### Mass spectrometry analysis

10^6^ cells were subjected to lipid extraction with 1.5 mL methanol/chloroform (2:1, *v:v*)^72^. The extraction solvent contained d_7_-sphingosine (d_7_-Sph), C17-ceramide, and C16-d_31_-sphingomyelin (all from Avanti Polar Lipids) as internal standards. Sample analysis was carried out by liquid chromatography tandem-mass spectrometry (LC-MS/MS) using either a TQ 6490 mass spectrometer (for Sph) or a QTOF 6530 mass spectrometer (for ceramides and sphingomyelins) (both from Agilent Technologies, Waldbronn, Germany) operating in the positive electrospray ionization mode (ESI+). The following selected reaction monitoring (SRM) transitions were used for quantification: *m/z* 300.3 → 282.3 for Sph and *m/z* 307.3 → 289.3 for d_7_-Sph. The precursor ions of ceramide or sphingomyelin species (differing in the lengths of their fatty acid chains) were cleaved into the fragment ions *m/z* 264.270 (for ceramide) or *m/z* 184.074 (for sphingomyelin), respectively^73^. Quantification was performed with Mass Hunter Software (Agilent Technologies).

### Electron microscopy

The samples were fixed with glutaraldehyde (2.5% in 100 mM phosphate buffer). Cells were then contrasted with 1% osmium tetroxide, 1% thiocarbohydrazide, 1.5% potassium ferrocyanide, and 1% uranyl acetate in water and were then dehydrated in an ascending ethanol row (50%, 70%, 96%, and 3 × 100%, 10 min each) and stepwise (EPON/EtOH 1:1 and 2:1 each for 2 h, EPON overnight) flat-embedded in EPON resin. Subsequently, EPON-embedded samples were polymerized for 24 h at 60 °C. After removal of the glass slide with 40% aqueous hydrofluoric acid, the EPON disk, containing the cells of interest, was removed from the dish, mounted on EPON resin blocks, and processed for ultramicrotomy. Serial sections were cut with a Leica EM UC7 ultramicrotome (Leica Microsystems) set to a thickness of 55 nm. A final post staining of the ultrathin sections was perfomed for 6 min on a drop of 1% uranylacetate followed by 1% lead citrate for 3 min in aqueous solution.

Transmission electron microscopy (TEM) was conducted on a Jeol 1400FPlus (Tokyo, Japan) instrument at 120 kV with magnifications as indicated. Digital images were acquired with a 4096×4096-pixel complementary metal-oxide semiconductor (CMOS) camera (TemCam-F416, TVIPS; Gauting, Germany). Postprocessing of the resulting 16-bit TIFF image files was performed with Fiji software version 1.52e (Image J). Pixel noise was averaged by a gaussian blur algorithm. A background resulting from inhomogeneous illumination was removed by implementing the “rolling ball” procedure, and the contrast was adjusted with histogram normalization.

### Statistical analysis

If not mentioned otherwise, data are expressed as arithmetic mean ± SEM and *n* represents the number of mice. Student’s *t*-test (two-tailed, if not indicated otherwise) in case of normal distribution or in case of multiple comparisons ANOVA, were used to detect statistically significant differences. *p*-Values of 0.05 or less were considered statistically significant. Statistical analyses and graphical presentations were computed with Graph Pad Prism software (Graph Pad, La Jolla, USA).

### Reporting summary

Further information on research design is available in the Nature Research Reporting Summary linked to this article.

## Supplementary information

Supplementary Information

Reporting Summary

## Data Availability

The authors declare that data that support the findings of this study are available from the corresponding author upon reasonable request. Microarray data are deposited in the Gene Expression Omnibus (GEO) under the accession code GSE142175.

## Source Data

Source Data
