# Supplementary material for: Acid ceramidase of macrophages traps herpes simplex virus in multivesicular bodies and protects from severe disease
Source: Nat Commun. 2020 Mar 12;11:1338. doi: 10.1038/s41467-020-15072-8 (PMC7067866; doi:10.1038/s41467-020-15072-8)
Supplement: Supplementary file 1 — Supplementary Information [file 41467_2020_15072_MOESM1_ESM.pdf]

Acid ceramidase of macrophages traps herpes simplex virus in multivesicular bodies and protects from severe disease

Lang et al.

Supplementary Information

Chemical synthesis of *ω*-azido-sphingosine

General Experimental Information

Commercially available chemical reagents, purchased from *Sigma-Aldrich*, *Alfa Aesar*, *TCI* and *ACROS*, were used as received without further purification. All solvents were distilled before usage and moisture-sensitive reactions were performed under nitrogen atmosphere. Analytical thin-layer chromatography (TLC) was performed using silica gel coated aluminum plates with a thickness of 0.2 mm (*Macherey-Nagel*). The compounds were visualized with a potassium permanganate stain solution containing 1.50 g $KMnO_4$, 10.0 g K_2CO_3 and 100 mg NaOH in 200 mL H_2O. Liquid column chromatography purification was performed with silica gel 60 (40–63 µm mesh, *Macherey-Nagel*).

Nuclear magnetic resonance (NMR) spectra were recorded on a *Bruker* Avance III HD 400 at 295 K. Chemical shifts (δ) are given in parts per million (ppm) with respect to the solvent residual proton signals (δ($CDCl_3$) = 7.26 ppm, δ(CD_3OD) = 3.31 ppm) for ^1H or the resonance signals (δ($CDCl_3$) = 77.16 ppm, δ(CD_3OD) = 49.00 ppm) for ^{13}C. Coupling constants (*J*) are reported in Hertz (Hz) and the multiplicity is abbreviated as s (singlet), d (doublet), t (triplet), m (multiplet), dd (doublet of doublets), br d (broad doublet) etc. Signal assignment was performed with additional information of DEPT135, (^1H,^1H)-COSY, (^1H,^{13}C)-HSQC and (^1H,^{13}C)-HMBC. Atom numbers do not refer to the IUPAC nomenclature.

High resolution mass spectrometry (HRMS) was performed with a *Bruker* Daltonics micrOTOF-Q III (electrospray ionization, ESI) instrument.

Abbreviations

Boc, *tert*-butoxycarbonyl; CyH, cyclohexane; DMF, *N*,*N*-dimethylformamide; EtOAc, ethyl acetate; Grubbs 2nd, (1,3-Bis(2,4,6-trimethylphenyl)-2-imidazolidinylidene)-dichloro(phenylmethylene)(tricyclohexylphosphine)ruthenium; MeOH, methanol; NEt_3, triethylamine; rt, room temperature; TBAF, tetra-*n*-butylammonium fluoride; TBS, *tert*-butyldimethylsilyl-; TFA, trifluoroacetic acid; THF, tetrahydrofuran.

Chemical synthesis of *ω*-azido-sphingosine

Synthesis route of *ω*-azido-sphingosine:

The sphingoid backbone was obtained by olefin cross-metathesis reaction using Grubbs catalyst 2nd generation, which is a known method for the synthesis of various sphingolipid derivatives[1,2]. An azide-tagged sphingosine analogue with a C_{14}-backbone has already been described by Garrido *et al.* in 2015[3]. Here we synthesized a more natural C_{18} long-chain base, starting from the building blocks *tert*-butyl ((2*S*,3*R*)-1-((*tert*-butyldimethylsilyl)oxy)-3-hydroxypent-4-en-2-yl)carbamate (**1**) and 15-bromopentadec-1-ene (**2**). The syntheses of the allylic alcohol (**1**) and the brominated alkene (**2**) were performed according to literature[1,2]. After metathesis reaction, the desired *E*-configurated alkene *tert*-butyl ((2*S*,3*R*,*E*)-18-bromo-1-((*tert*-butyldimethylsilyl)oxy)-3-hydroxyoctadec-4-en-2-yl)carbamate (**3**) was isolated in 50% yield by column chromatography. Nucleophilic substitution of the terminal bromide with sodium azide in *N*,*N*-dimethylformamide afforded *tert*-butyl ((2*S*,3*R*,*E*)-18-azido-1-((*tert*-butyldimethylsilyl)oxy)-3-hydroxyoctadec-4-en-2-yl)carbamate (**4**) in an excellent yield. Subsequent cleavage of the silyl ether using tetra-*n*-butylammonium fluoride in tetrahydrofuran gave diol *tert*-butyl ((2*S*,3*R*,*E*)-18-azido-1,3-dihydroxyoctadec-4-en-2-yl)carbamate (**5**) in quantitative yield. In the final step, a dichloromethane solution of the carbamate was treated with trifluoroacetic acid to provide *ω*-azido-sphingosine (**6**) in 61% yield. All isolated compounds were fully characterized by a combination of ^{1}H- and ^{13}C-NMR spectroscopy and HRMS.

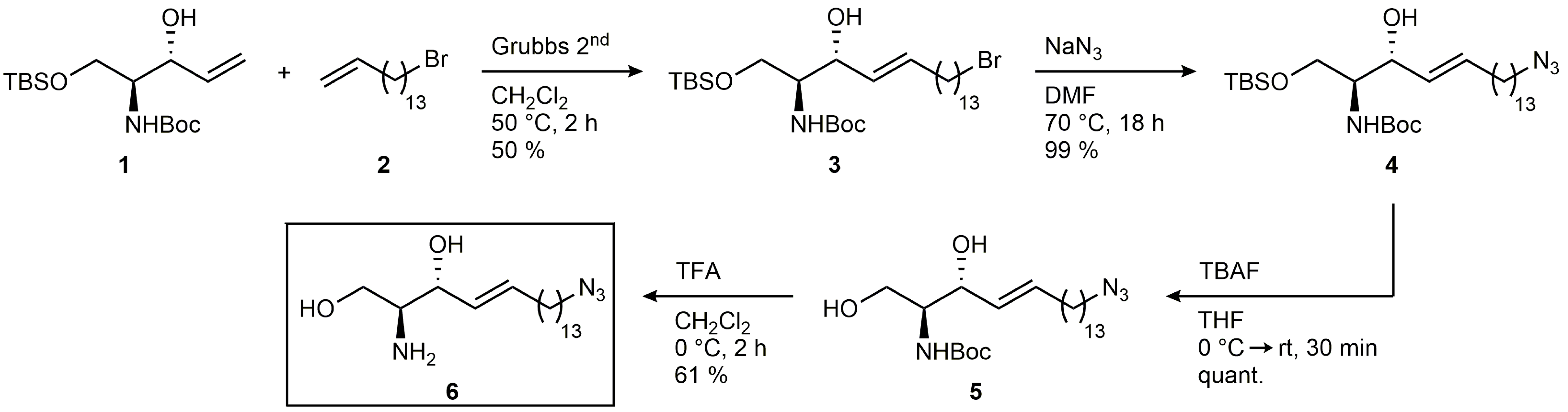

References

1. Yamamoto, T., Hasegawa, H., Hakogi, T. & Katsumura, S. Versatile synthetic method for sphingolipids and functionalized sphingosine derivatives via olefin cross metathesis. *Org Lett* 8, 5569-5572 (2006).

2. Qu, W., Ploessl, K., Truong, H., Kung, M.P. & Kung, H.F. Iodophenyl tagged sphingosine derivatives: synthesis and preliminary biological evaluation. *Bioorg Med Chem Lett* 19, 3382-3385 (2009).

3. Garrido, M., Abad, J.L., Fabrias, G., Casas, J. & Delgado, A. Azide-tagged sphingolipids: new tools for metabolic flux analysis. *Chembiochem* 16, 641-650 (2015).

Chemical synthesis of *ω*-azido-sphingosine

Experimental Procedures

tert-Butyl ((*2S*,3*R*,*E*)-18-bromo-1-((*tert*-butyldimethylsilyl)oxy)-3-hydroxyoctadec-4-en-2-yl)carbamate (3)

To a solution of allylic alcohol **1** (2.00 g, 6.03 mmol, 1.00 eq.) and alkene **2** (6.98 g, 24.1 mmol, 4.00 eq.) in dry CH_2Cl_2 (50 mL) was added Grubbs catalyst 2nd generation (154 mg, 181 µmol, 0.03 eq.) at rt. The reaction mixture was stirred at 50 °C for 2 h. The solvent was removed under reduced pressure and the residue was purified by column chromatography on silica gel (CyH:EtOAc, 1:0 to 9:1 v/v) to give **3** (1.80 g, 3.03 mmol, 50 %) as a colourless, waxy solid.

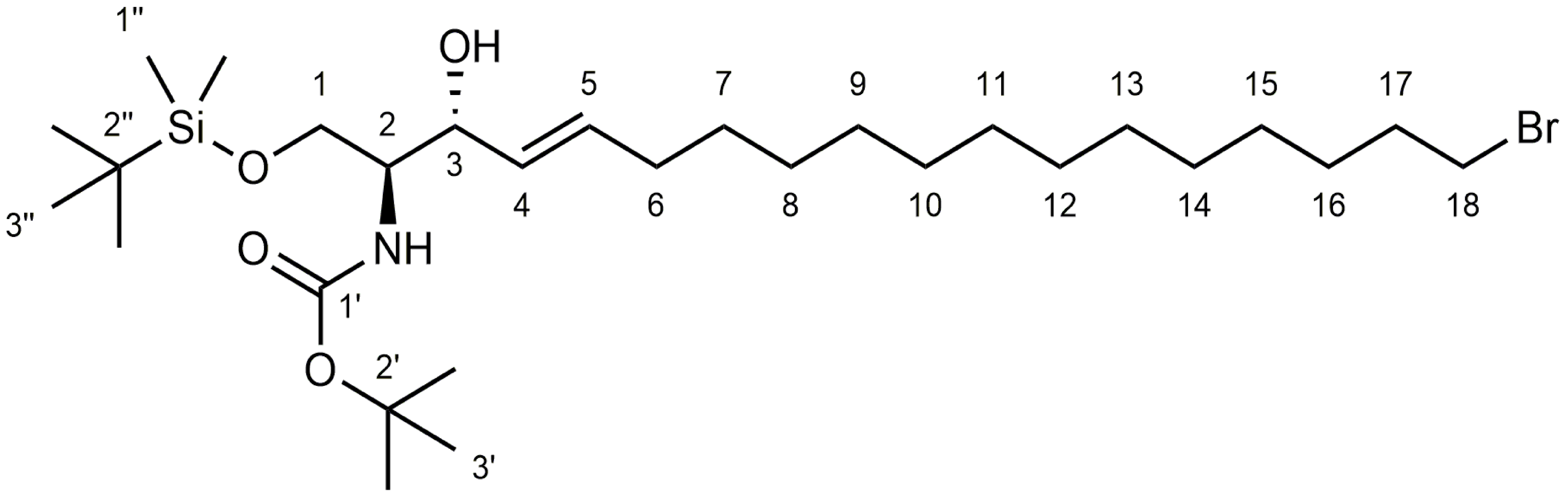

TLC (CyH:EtOAc, 9:1 v/v): R_f = 0.25; **^{1}H NMR** (400 MHz, $CDCl_3$): δ 5.74 (dtd, $^3J_{5,4}$ = 15.4 Hz, $^3J_{5,6}$ = 6.8 Hz, $^4J_{5,3}$ = 1.3 Hz, 1H, *H*-5), 5.49 (ddt, $^3J_{4,5}$ = 15.4 Hz, $^3J_{4,3}$ = 5.9 Hz, $^4J_{4,6}$ = 1.3 Hz, 1H, *H*-4), 5.23 (br d, $^3J_{NH,2}$ = 8.2 Hz, 1H, N*H*), 4.16–4.20 (m, 1H, *H*-3), 3.93 (dd, $^2J_{1,1}$ = 10.3 Hz, $^3J_{1,2}$ = 3.0 Hz, 1H, *H*-1), 3.74 (br dd, $^2J_{1,1}$ = 10.3 Hz, $^3J_{1,2}$ = 2.6 Hz, 1H, *H*-1), 3.54–3.58 (m, 1H, *H*-2), 3.40 (t, $^3J_{18,17}$ = 6.9 Hz, 2H, *H*-18), 3.33 (br d, $^3J_{OH,3}$ = 7.5 Hz, 1H, O*H*), 2.02–2.07 (m, 2H, *H*-6), 1.81–1.88 (m, 2H, *H*-17), 1.44 (s, 9H, *H*-3'), 1.25–1.42 (m, 20H, *H*-7–16), 0.89 (s, 9H, *H*-3''), 0.06 (s, 3H, *H*-1''), 0.06 (s, 3H, *H*-1''); **^{13}C NMR** (100 MHz, $CDCl_3$): δ 155.9 (*C*-1'), 133.2 (*C*-5), 129.6 (*C*-4), 79.5 (*C*-2'), 74.8 (*C*-3), 63.6 (*C*-1), 54.6 (*C*-2), 34.2 (*C*-18), 33.0 (*C*-17), 32.4 (*C*-6), 29.8, 29.7, 29.7, 29.6, 29.6, 29.3, 28.9, 28.3 (overall 10C, *C*-7–16), 28.5 (3C, *C*-3'), 25.9 (3C, *C*-3''), 18.2 (*C*-2''), −5.5 (*C*-1''), −5.5 (*C*-1''); **HRMS** (m/z): $[M+Na]^+$ calcd. for $C_{29}H_{58}BrNNaO_4Si$, 614.3211; found, 614.3223.

Chemical synthesis of *ω*-azido-sphingosine

***tert*-Butyl ((2*S*,3*R*,*E*)-18-azido-1-((*tert*-butyldimethylsilyl)oxy)-3-hydroxyoctadec-4-en-2-yl)carbamate (4)**

To a solution of bromide **3** (1.00 g, 1.69 mmol, 1.00 eq.) in DMF (20 mL) was added NaN_3 (329 mg, 5.06 mmol, 3.00 eq.). The reaction mixture was stirred at 70 °C for 18 h, cooled to rt and then H_2O (100 mL) was added. After the extraction with EtOAc (5 x 30 mL), the combined organic phases were washed with brine (20 mL) and dried ($MgSO_4$). The solvents were removed under reduced pressure to give **4** (930 mg, 1.68 mmol, 99 %) as a colourless, waxy solid.

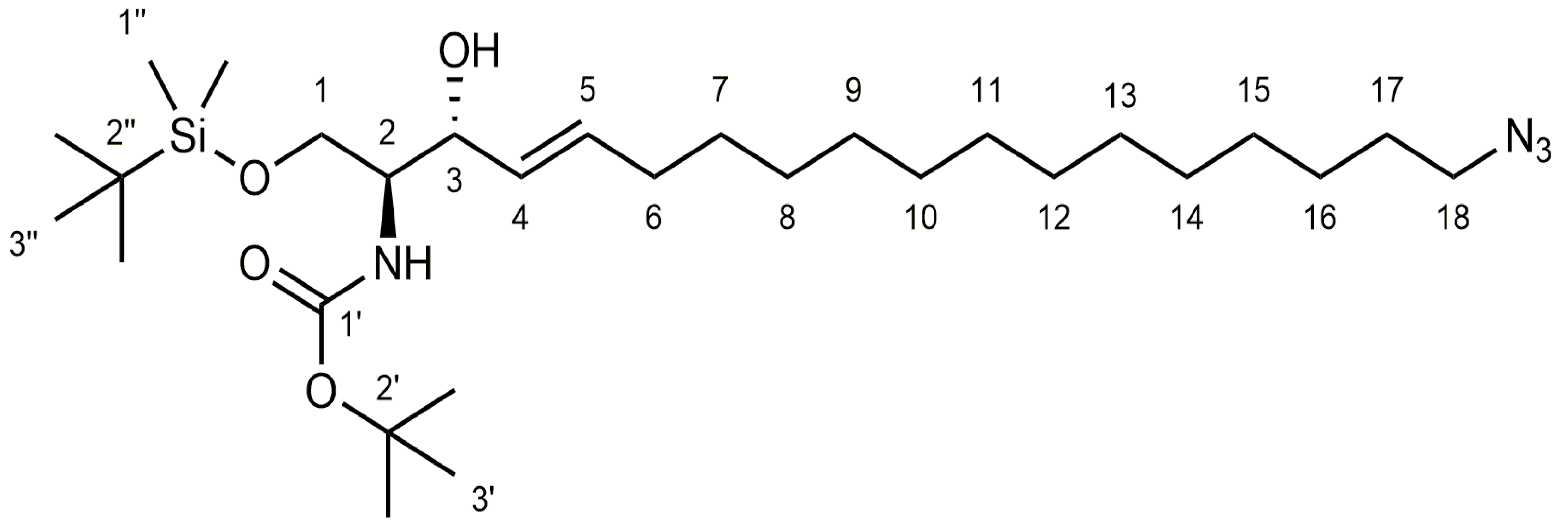

TLC (CyH:EtOAc, 9:1 v/v): R_f = 0.25; **^{1}H NMR** (400 MHz, $CDCl_3$): δ 5.74 (dtd, $^3J_{5,4}$ = 15.3 Hz, $^3J_{5,6}$ = 6.8 Hz, $^4J_{5,3}$ = 1.2 Hz, 1H, *H*-5), 5.49 (ddt, $^3J_{4,5}$ = 15.3 Hz, $^3J_{4,3}$ = 5.9 Hz, $^4J_{4,6}$ = 1.3 Hz, 1H, *H*-4), 5.23 (br d, $^3J_{NH,2}$ = 8.2 Hz, 1H, N*H*), 4.17–4.19 (m, 1H, *H*-3), 3.92 (dd, $^2J_{1,1}$ = 10.3 Hz, $^3J_{1,2}$ = 3.0 Hz, 1H, *H*-1), 3.74 (br dd, $^2J_{1,1}$ = 10.3 Hz, $^3J_{1,2}$ = 2.5 Hz, 1H, *H*-1), 3.54–3.57 (m, 1H, *H*-2), 3.33 (br s, 1H, O*H*), 3.24 (t, $^3J_{18,17}$ = 7.0 Hz, 2H, *H*-18), 2.01–2.07 (m, 2H, *H*-6), 1.55–1.62 (m, 2H, *H*-17), 1.44 (s, 9H, *H*-3'), 1.25–1.38 (m, 20H, *H*-7–16), 0.89 (s, 9H, *H*-3''), 0.06 (s, 3H, *H*-1''), 0.05 (s, 3H, *H*-1''); **^{13}C NMR** (100 MHz, $CDCl_3$): δ 155.9 (*C*-1'), 133.2 (*C*-5), 129.6 (*C*-4), 79.5 (*C*-2'), 74.8 (*C*-3), 63.6 (*C*-1), 54.6 (*C*-2), 51.6 (*C*-18), 32.4 (*C*-6), 29.8, 29.7, 29.7, 29.6, 29.6, 29.3, 29.3, 26.8 (overall 10C, *C*-7–16), 29.0 (*C*-17), 28.5 (3C, *C*-3'), 25.9 (3C, *C*-3''), 18.2 (*C*-2''), −5.5 (*C*-1''), −5.5 (*C*-1''); **HRMS** (m/z): $[M+Na]^+$ calcd. for $C_{29}H_{58}N_4NaO_4Si$, 577.4120; found, 577.4126.

Chemical synthesis of *ω*-azido-sphingosine

***tert*-Butyl ((2*S*,3*R*,*E*)-18-azido-1,3-dihydroxyoctadec-4-en-2-yl)carbamate (5)**
To a solution of silyl ether **4** (1.84 g, 3.32 mmol, 1.00 eq.) in dry THF (35 mL) was added TBAF (1 M in THF, 3.98 mL, 3.98 mmoL, 1.20 eq.) at 0 °C. The ice bath was removed and the reaction mixture was stirred at rt for 30 min. After the addition of H_2O (50 mL) and brine (50 mL), the aqueous layer was extracted with EtOAc (5 x 50 mL). The combined organic phases were washed with brine (25 mL), dried ($MgSO_4$) and the solvents were removed under reduced pressure. The residue was purified by column chromatography on silica gel (CyH:EtOAc, 2:1 v/v) to give **5** (1.46 g, 3.31 mmol, quant.) as a colourless, waxy solid.

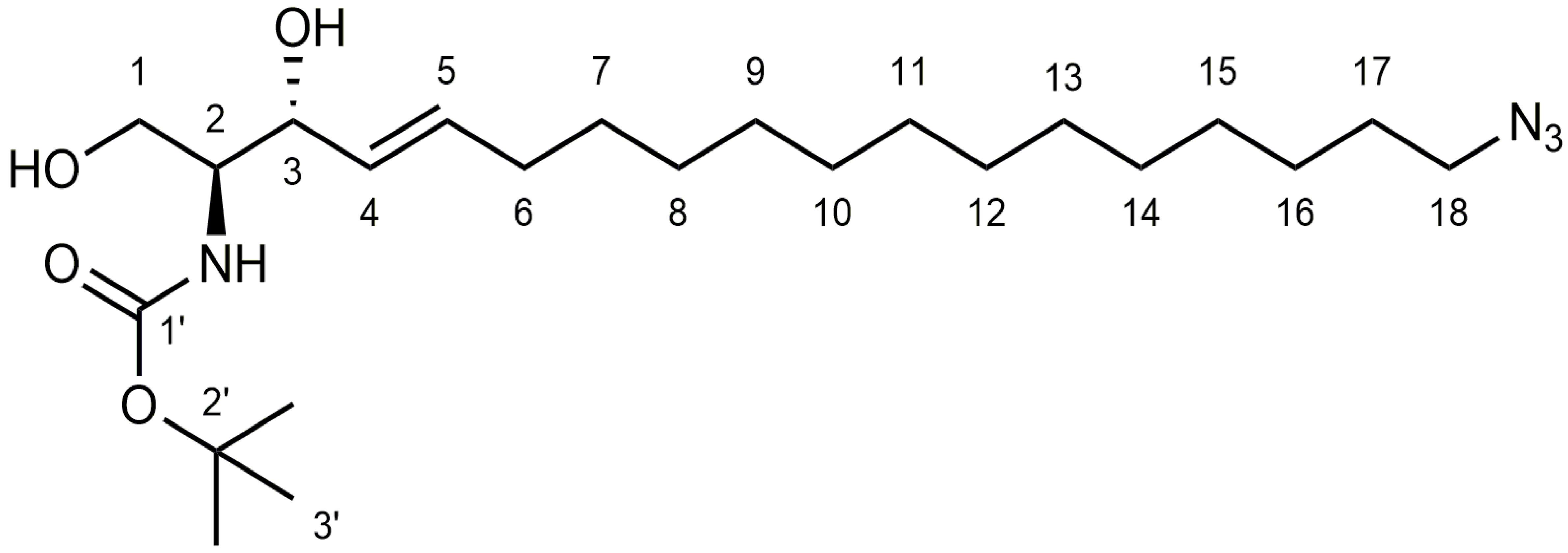

TLC (CyH:EtOAc, 2:1 v/v): R_f = 0.20; **^{1}H NMR** (400 MHz, $CDCl_3$): δ 5.75 (dtd, $^3J_{5,4}$ = 15.3 Hz, $^3J_{5,6}$ = 6.8 Hz, $^4J_{5,3}$ = 1.2 Hz, 1H, *H*-5), 5.50 (ddt, $^3J_{4,5}$ = 15.3 Hz, $^3J_{4,3}$ = 6.4 Hz, $^4J_{4,6}$ = 1.2 Hz, 1H, *H*-4), 5.34 (br d, $^3J_{NH,2}$ = 7.9 Hz, 1H, N*H*), 4.27–4.29 (m, 1H, *H*-3), 3.91 (dd, $^2J_{1,1}$ = 11.3 Hz, $^3J_{1,2}$ = 3.7 Hz, 1H, *H*-1), 3.68 (dd, $^2J_{1,1}$ = 11.3 Hz, $^3J_{1,2}$ = 3.6 Hz, 1H, *H*-1), 3.56–3.59 (m, 1H, *H*-2), 3.24 (t, $^3J_{18,17}$ = 7.0 Hz, 2H, *H*-18), 3.00 (br s, 2H, 2 x O*H*), 2.01–2.06 (m, 2H, *H*-6), 1.54–1.62 (m, 2H, *H*-17), 1.43 (s, 9H, *H*-3'), 1.24–1.38 (m, 20H, *H*-7–16); **^{13}C NMR** (100 MHz, $CDCl_3$): δ 156.4 (*C*-1'), 134.2 (*C*-5), 129.0 (*C*-4), 79.9 (*C*-2'), 74.8 (*C*-3), 62.7 (*C*-1), 55.5 (*C*-2), 51.6 (*C*-18), 32.4 (*C*-6), 29.7, 29.7, 29.7, 29.6, 29.6, 29.3, 29.3, 29.2, 26.8 (overall 10C, *C*-7–16), 28.9 (*C*-17), 28.5 (3C, *C*-3'); **HRMS** (m/z): $[M+Na]^+$ calcd. for $C_{23}H_{44}N_4NaO_4$, 463.3255; found, 463.3250.

Chemical synthesis of *ω*-azido-sphingosine

(2*S*,3*R*,*E*)-2-Amino-18-azidooctadec-4-ene-1,3-diol / *ω*-azido-sphingosine (6)

To a solution of carbamate **5** (900 mg, 2.04 mmol) in CH_2Cl_2 (20 mL) was added TFA (4.72 mL) at 0 °C. The reaction mixture was stirred at 0 °C for 2 h and subsequently treated with H_2O (100 mL) and 1 M aq. NaOH (100 mL). After the extraction with EtOAc (10 x 50 mL), the combined organic phases were washed with brine (10 mL), dried ($MgSO_4$) and concentrated under reduced pressure. The residue was purified by column chromatography on silica gel (CH_2Cl_2:MeOH, 9:1 v/v) to give **6** (423 mg, 1.24 mmol, 61 %) as a colourless, waxy solid.

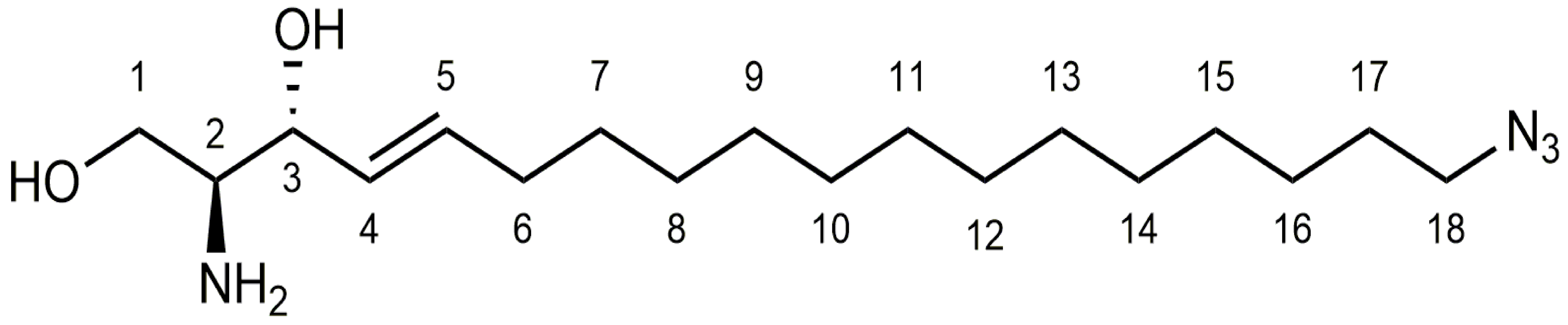

TLC (CH_2Cl_2:MeOH:NEt_3, 10:1:0.1 v/v): R_f = 0.08; **^{1}H NMR** (400 MHz, CD_3OD): δ 5.76 (dtd, $^3J_{5,4}$ = 15.3 Hz, $^3J_{5,6}$ = 6.8 Hz, $^4J_{5,3}$ = 1.0 Hz, 1H, *H*-5), 5.49 (ddt, $^3J_{4,5}$ = 15.3 Hz, $^3J_{4,3}$ = 7.3 Hz, $^4J_{4,6}$ = 1.4 Hz, 1H, *H*-4), 4.02–4.06 (m, 1H, *H*-3), 3.70 (dd, $^2J_{1,1}$ = 11.0 Hz, $^3J_{1,2}$ = 4.4 Hz, 1H, *H*-1), 3.53 (dd, $^2J_{1,1}$ = 11.0 Hz, $^3J_{1,2}$ = 7.3 Hz, 1H, *H*-1), 3.28 (t, $^3J_{18,17}$ = 6.9 Hz, 2H, *H*-18), 2.85 (ddd, $^3J_{2,1}$ = 7.3 Hz, $^3J_{2,3}$ = 5.8 Hz, $^3J_{2,1}$ = 4.4 Hz, 1H, *H*-2), 2.06–2.11 (m, 2H, *H*-6), 1.55–1.62 (m, 2H, *H*-17), 1.30–1.44 (m, 20H, *H*-7–16); **^{13}C NMR** (100 MHz, CD_3OD): δ 135.6 (*C*-5), 130.3 (*C*-4), 74.2 (*C*-3), 63.2 (*C*-1), 58.1 (*C*-2), 52.4 (*C*-18), 33.4 (*C*-6), 30.8, 30.7, 30.7, 30.6, 30.4, 30.3, 30.3, 27.8 (overall 10C, *C*-7–16), 29.9 (*C*-17); **HRMS** (m/z): $[M+H]^+$ calcd. for $C_{18}H_{37}N_4O_2$, 341.2911; found, 341.2917.

Supplementary Figure 1

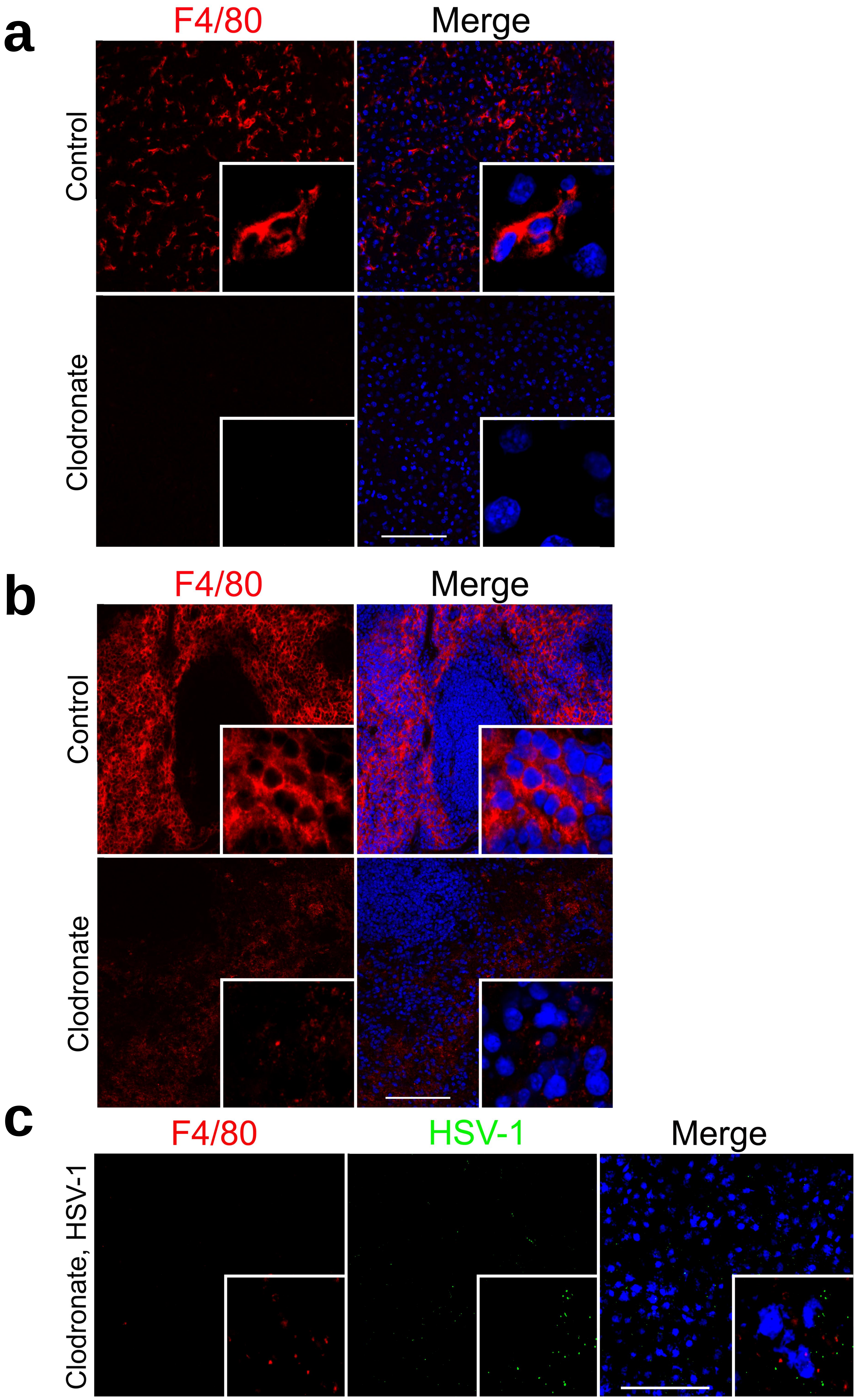

Supplementary Figure 1: Efficiency of macrophage depletion by clodronate

a&b: Immunofluorescence of livers (a) and spleens (b) from wild-type (WT) mice that were pretreated with control-liposomes and WT mice that were pretreated with clodronate-liposomes (day -3), infected with 6×10^6 tissue culture infection dose 50 ($TCID_{50}$) HSV-1 and analyzed after 24 h (n = 3, blue represents Hoechst staining, scale bar 100 µm). **c**: Immunofluorescence of livers from WT mice that were pretreated with clodronate-liposomes (day -3), infected with 8×10^7 $TCID_{50}$ HSV-1 and analyzed after 1 h (n = 3, blue represents Hoechst staining, scale bar 100 µm).

Supplementary Figure 2

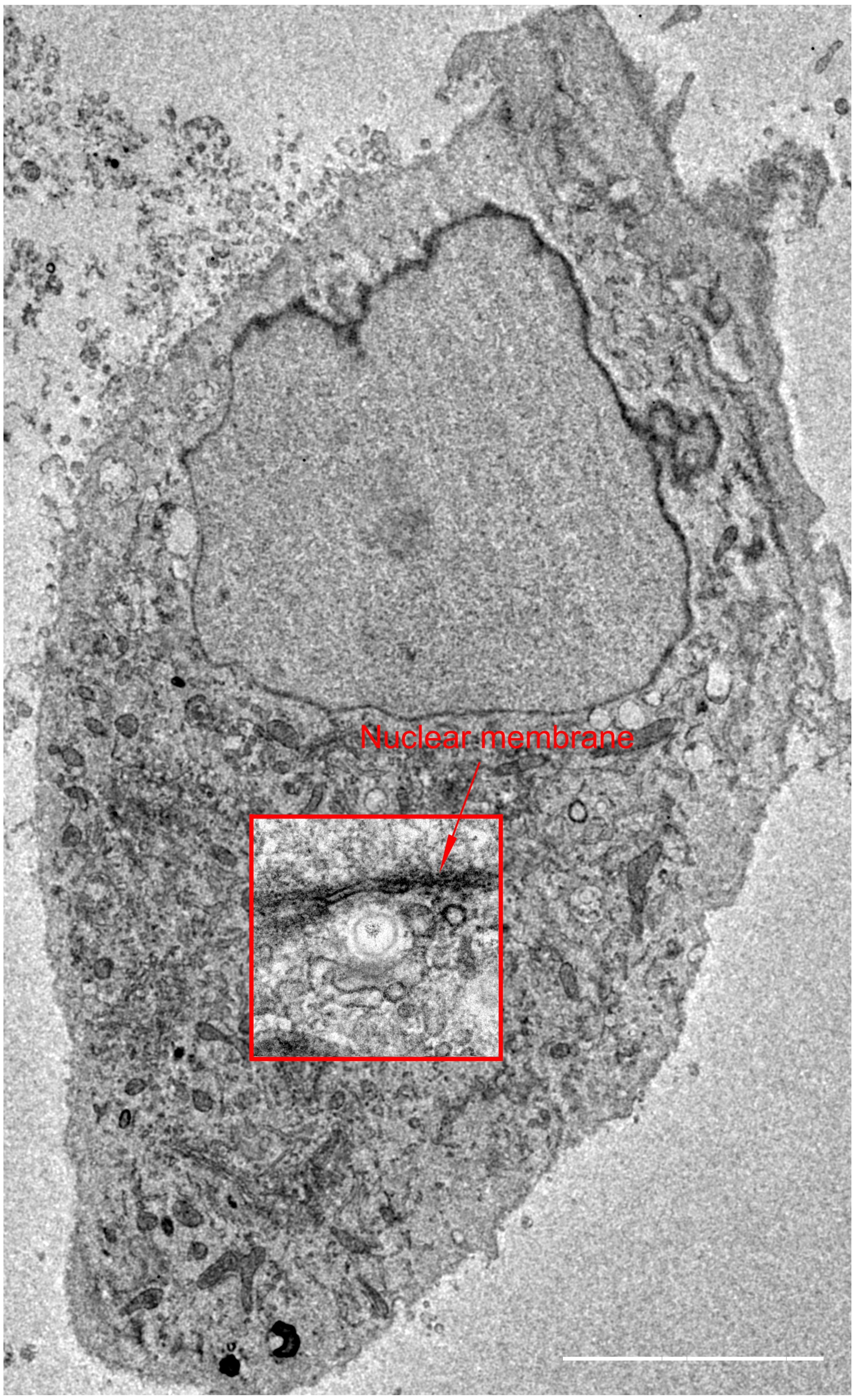

Supplementary Figure 2: Herpes simplex virus type 1 (HSV-1) infection of primary fibroblasts
Representative electron microscopy images of wild-type (WT) fibroblasts infected with HSV-1 (MOI 250) analyzed after 30 minutes (n = 106 images from three independent experiments, scale bar 5 µm). Detail shows HSV-1 close to the nucleus.

Supplementary Figure 3

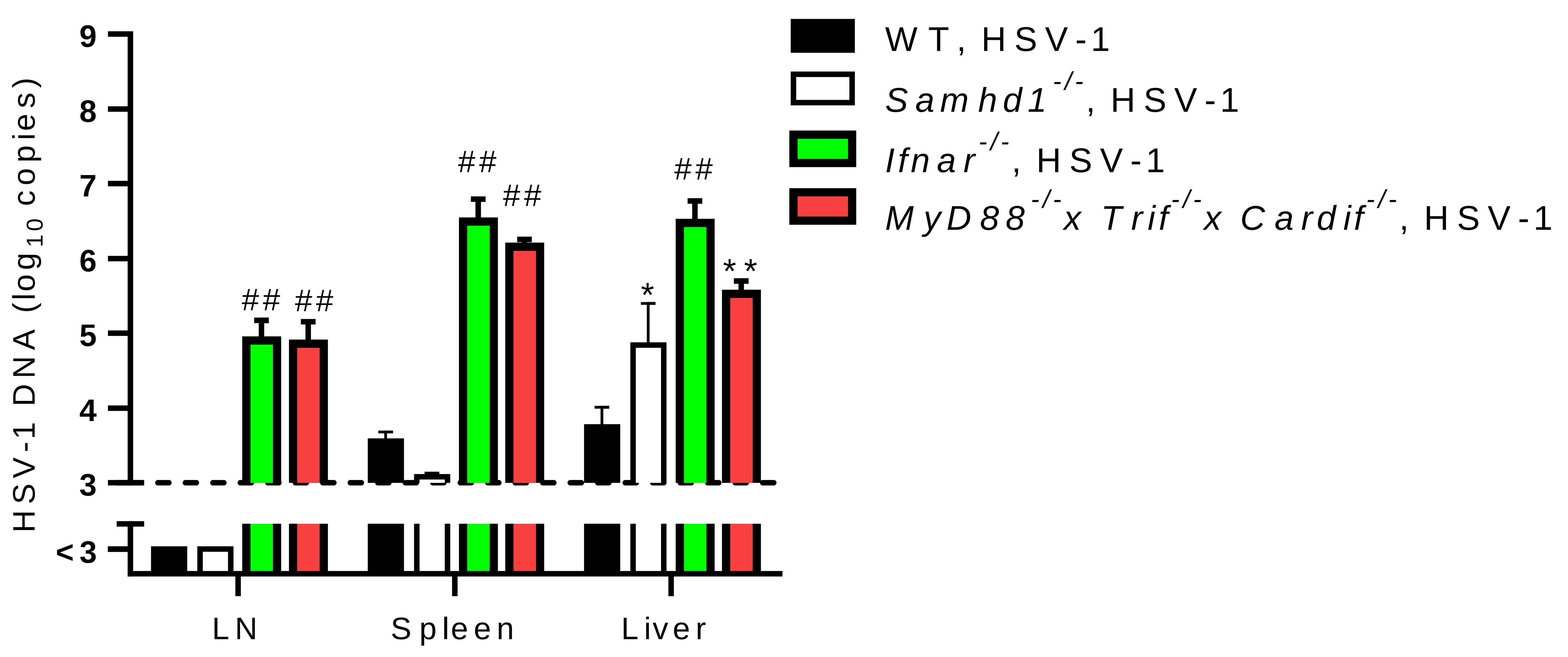

Supplementary Figure 3: Real-time polymerase chain reaction (RT-PCR) for herpes simplex virus type 1 (HSV-1) in mice with different innate immune deficiencies

RT-PCR of lymph nodes (LN), spleens and livers from wild-type (WT; n = 9), *Samhd1*$^{-/-}$ (n = 4), *Ifnar1*$^{-/-}$ (n = 4), and *MyD88*$^{-/-}$ *x Trif*$^{-/-}$ *x Cardif*$^{-/-}$ (n = 3) mice that were infected with 2×10^6 tissue culture infection dose 50 ($TCID_{50}$) HSV-1 and analyzed on day 3 (2way Anova [Tukey's multiple comparison]). All data are shown as mean +/- SEM. * equals $p \leq 0.05$, ** equals $p \leq 0.01$, # equals $p \leq 0.001$, ## equals $p \leq 0.0001$.

Supplementary Figure 4

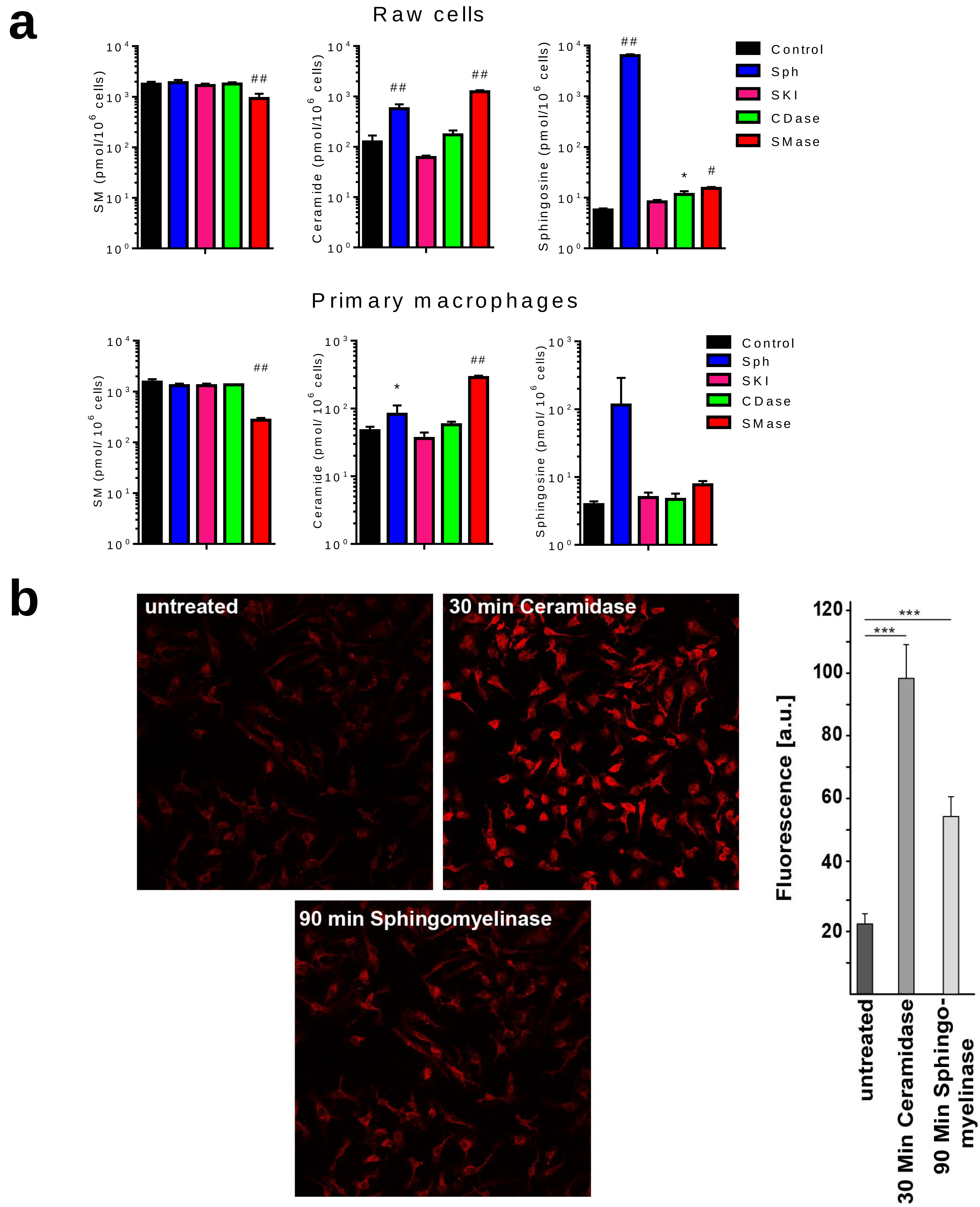

Supplementary Figure 4: Mass spectrometric analysis and immunofluorescence microscopy of sphingolipids after treatments in different cell lines

a: Mass spectrometric analysis of sphingolipids in Raw264.7 cells (n = 6-12) and bone marrow derived macrophages (BMDMs; n = 4) that were incubated for 30 minutes with 250 µM D-erythro-sphingosine (Sph), 100 µM of sphingosine kinase inhibitor (SKI), 250 U/L ceramidase (CDase) or 6.5 U/ml sphingomyelinase (SMase) and analyzed after 24 h (Raw264.7 cells, one-way Anova [Dunnett's /Kruskal-Wallis multiple comparison]) or 6h (BMDMs, one-way Anova [Dunnett's multiple comparison]). (**b**) BMDMs were treated for 30 minutes with 0.3 µg ceramidase, 90 minutes with 1.56 U / 250µL sphingomyelinase or left untreated (n = 3, one-way Anova [Tukey's multiple comparison]). All data are shown as mean +/- SD. * equals $p \leq 0.05$, ** equals $p \leq 0.01$, # equals $p \leq 0.001$, ## equals $p \leq 0.0001$.

Supplementary Figure 5

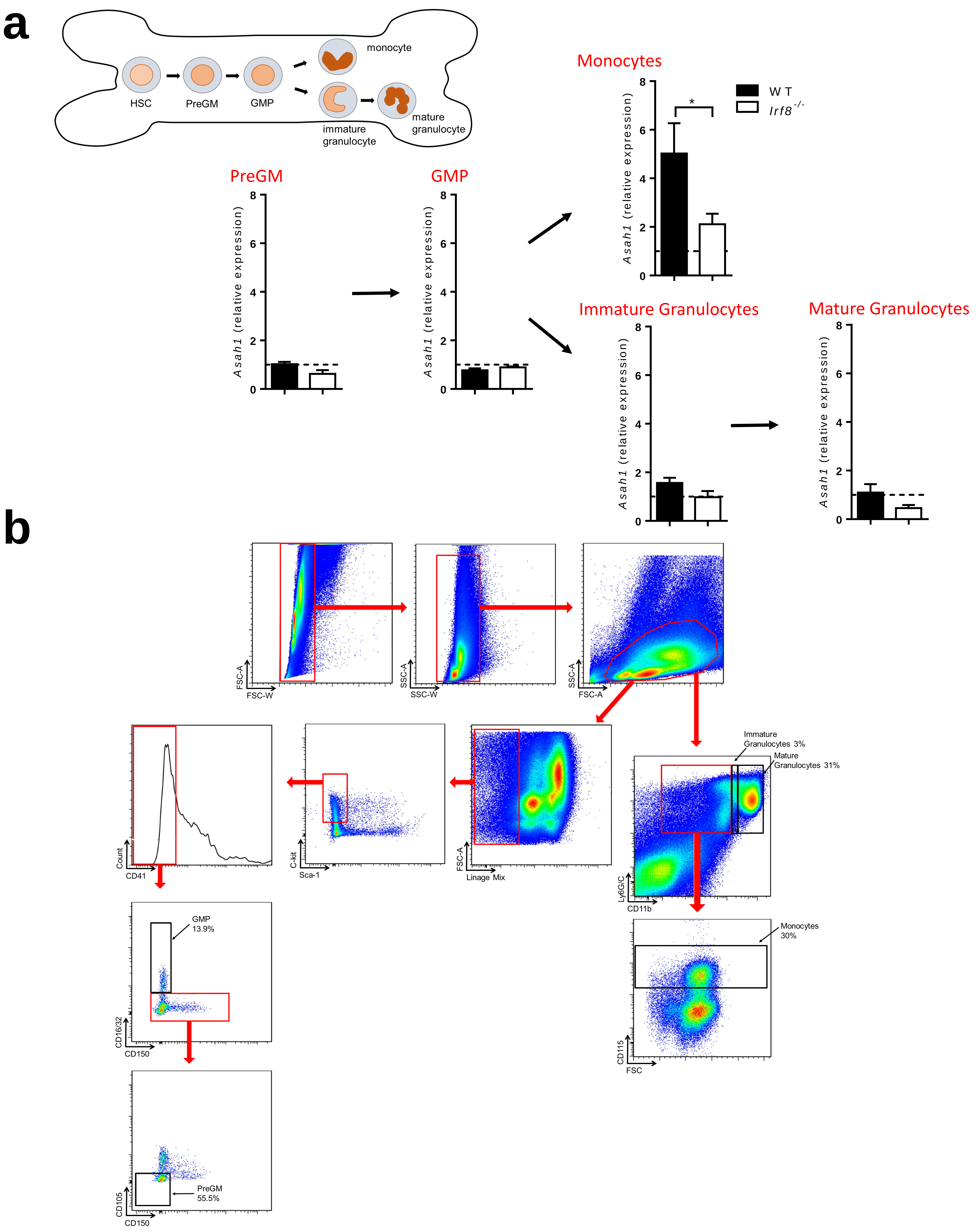

Supplementary Figure 5: Expression of *Asah1* in macrophages during development is dependent on IRF8

a: Real-time polymerase chain reaction (RT-PCR) results for *Asah1* mRNA expression of monocytes, granulocytes and precursor cells from the myeloid lineage, isolated from wild-type (WT) and IRF8 deficient mice (n = 5-8; one-tailed Student's *t*-test). **b**: Gating strategy for cell sorting. All data are shown as mean +/- SEM. * equals p ≤ 0.05, ** equals p ≤ 0.01, # equals p ≤ 0.001, ## equals p ≤ 0.0001.

Supplementary Figure 6

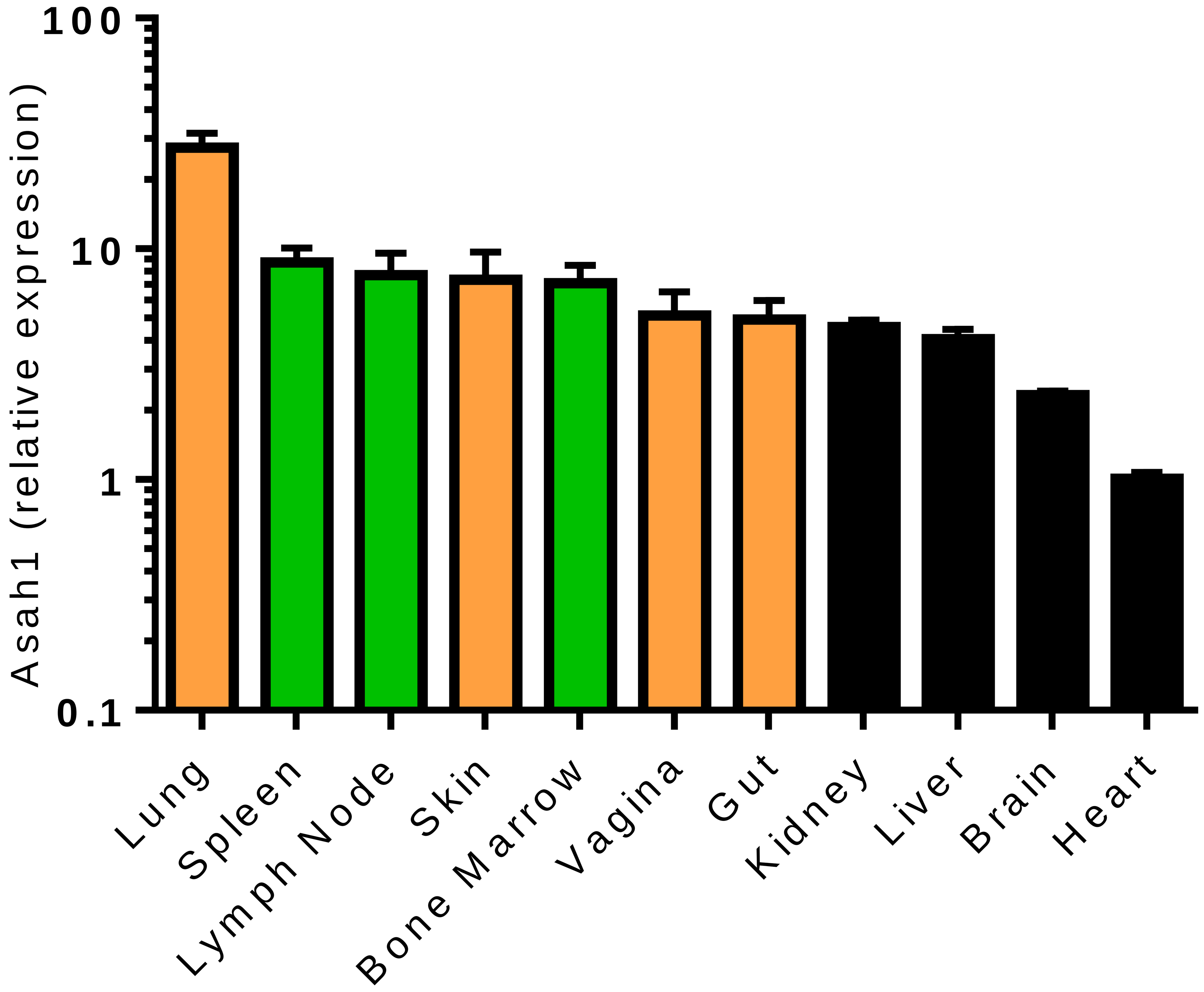

Supplementary Figure 6: Asah1 expression in different tissues
Real-time polymerase chain reaction (RT-PCR) for *Asah1* of the indicated organs, relative to heart, derived from naïve C57BL/6 mice (n = 4-5). All data are shown as mean +/- SEM.

Supplementary Figure 7

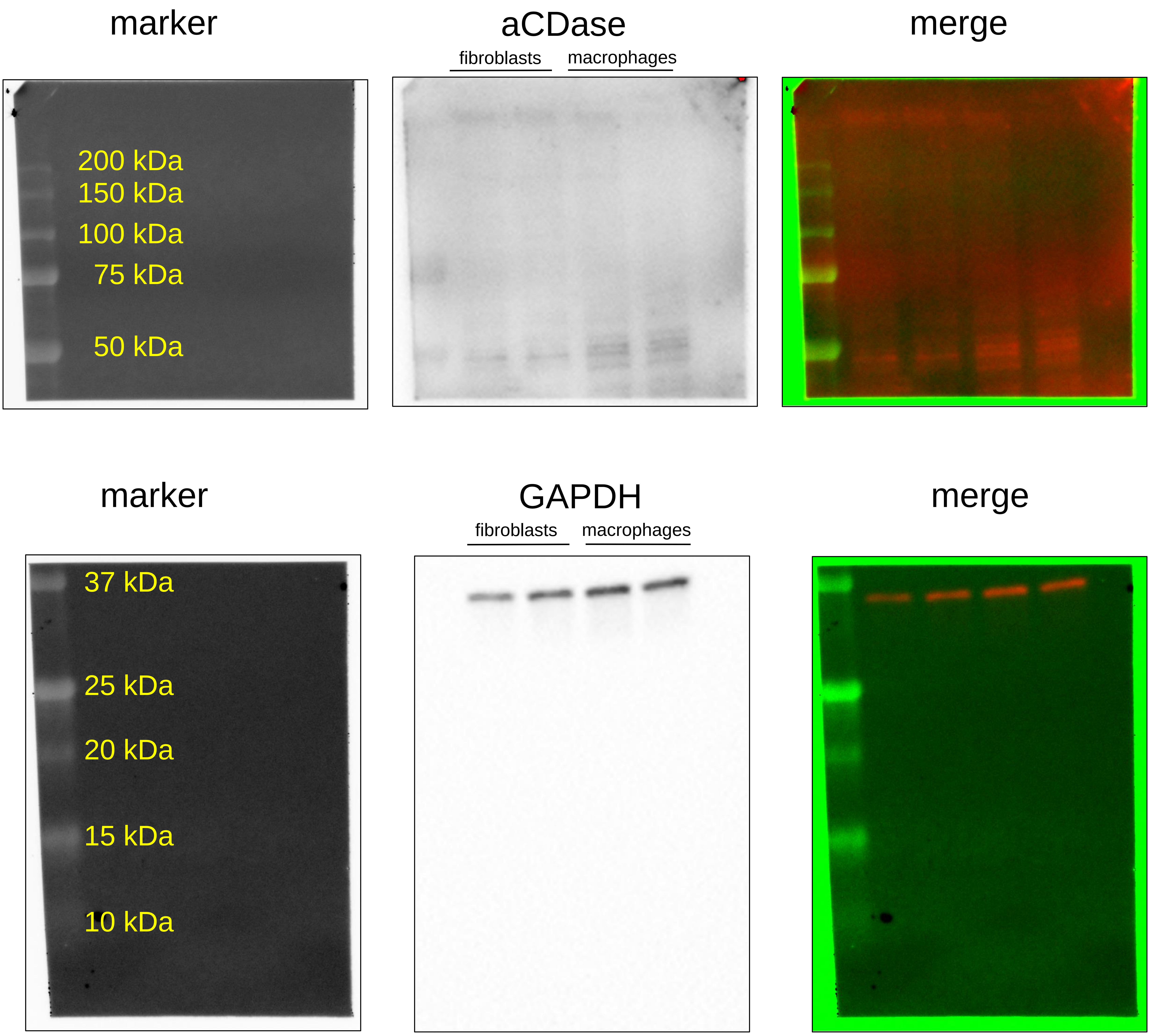

Supplementary Figure 7: Uncropped Western blots as shown in Figure 2h.

Supplementary Figure 8

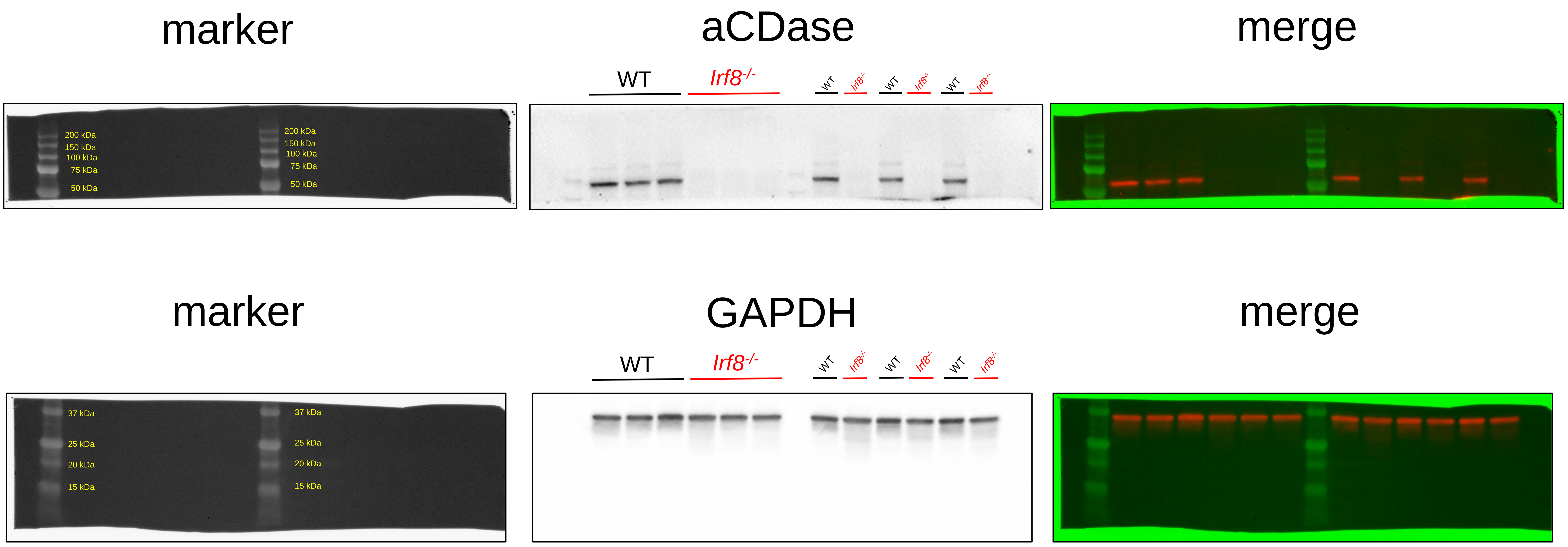

Supplementary Figure 8: Uncropped Western blots as shown in Figure 6d.

Supplementary Figure 9

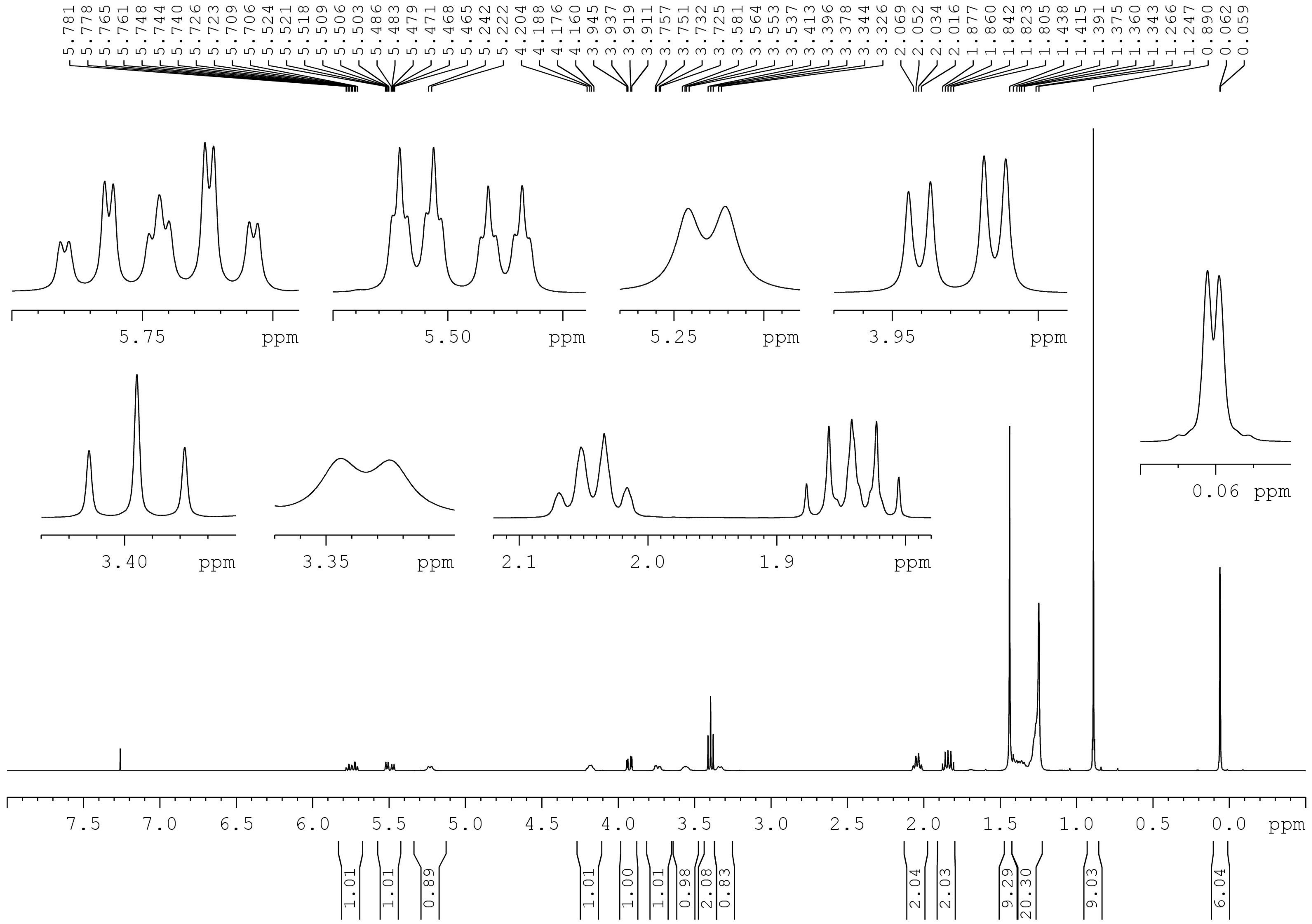

Supplementary Figure 9: ^{1}H NMR spectrum of **3** ($CDCl_3$, 400 MHz).

Supplementary Figure 10

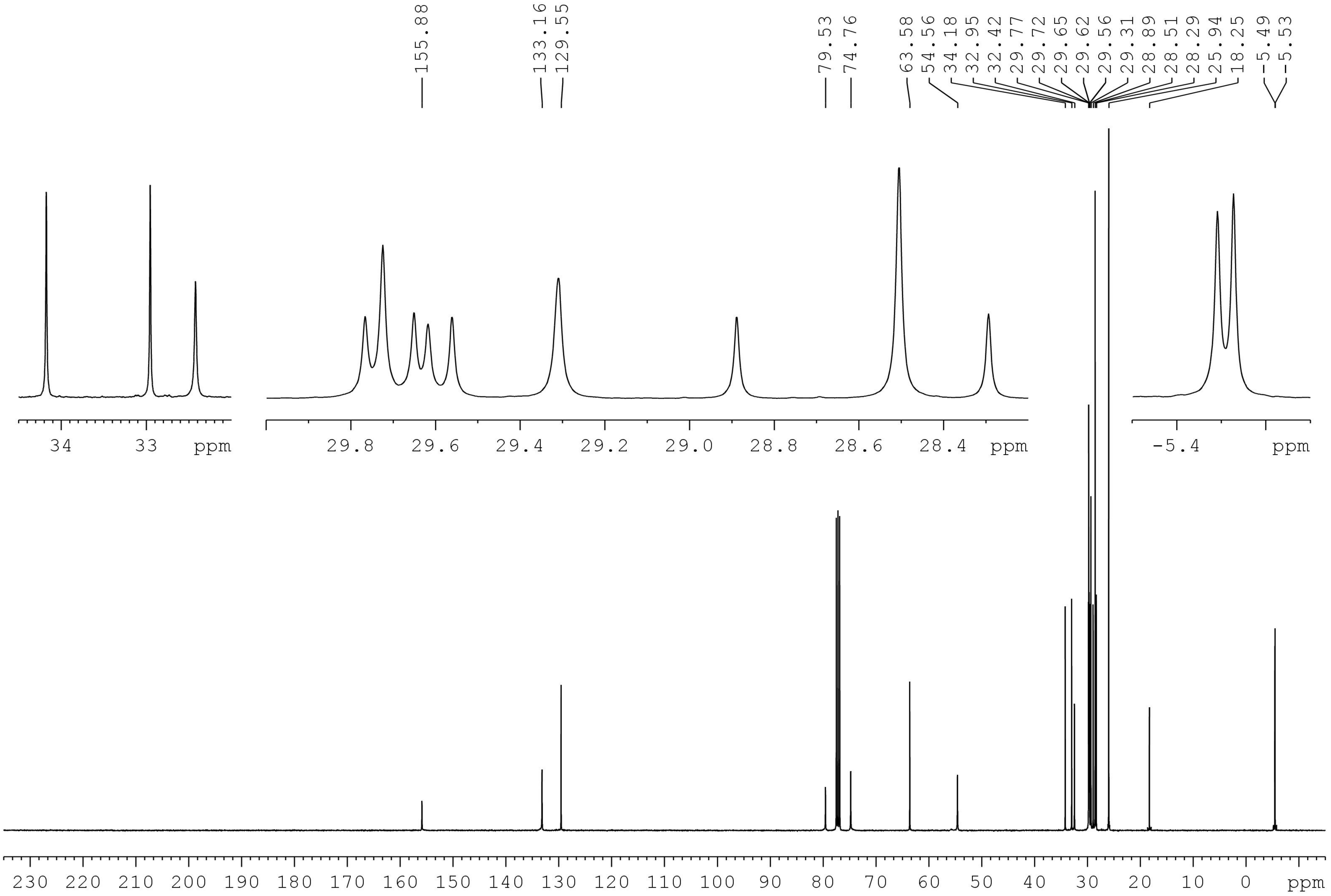

Supplementary Figure 10: ^{13}C NMR spectrum of **3** ($CDCl_3$, 100 MHz).

Supplementary Figure 11

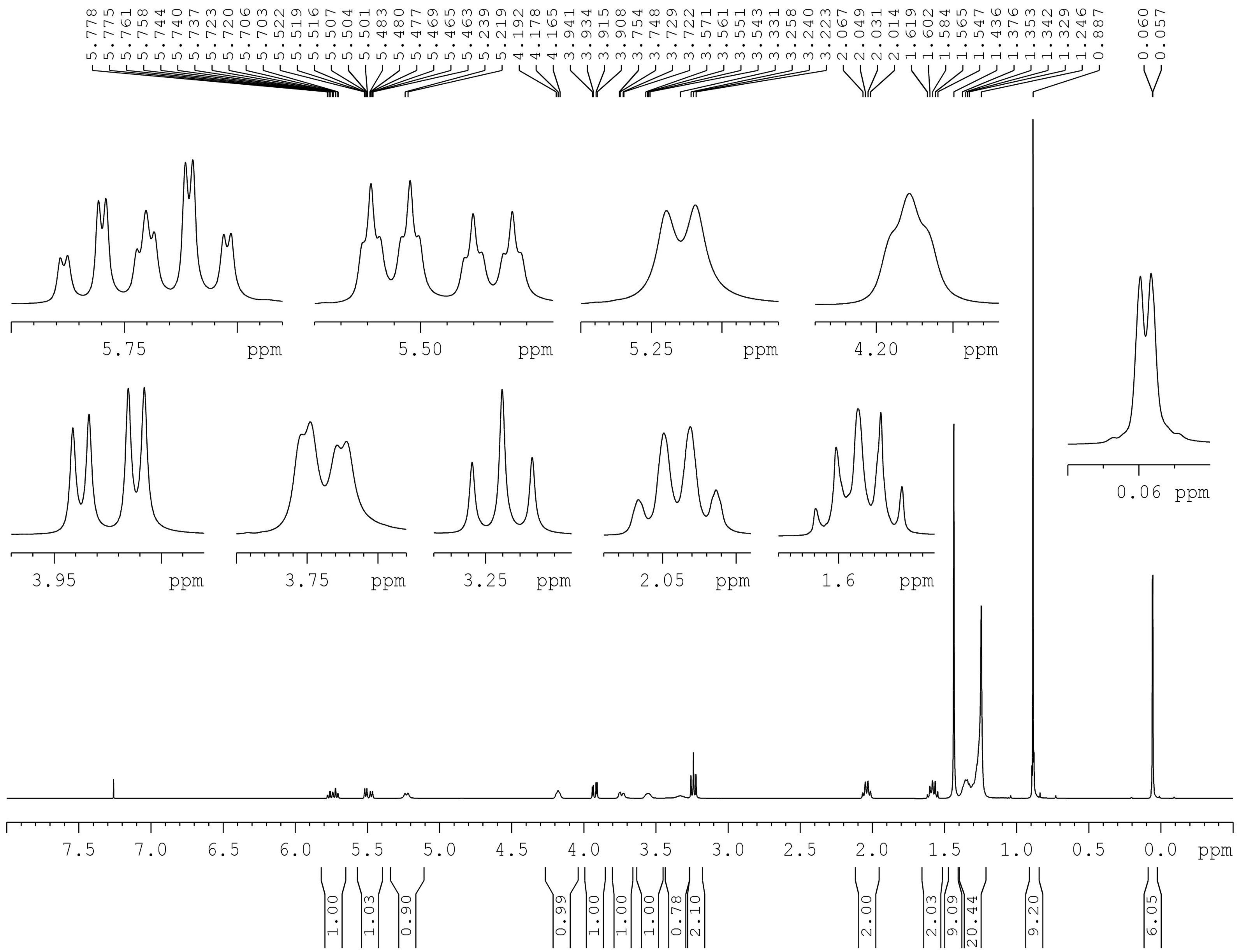

Supplementary Figure 11: ^{1}H NMR spectrum of **4** ($CDCl_3$, 400 MHz).

Supplementary Figure 12

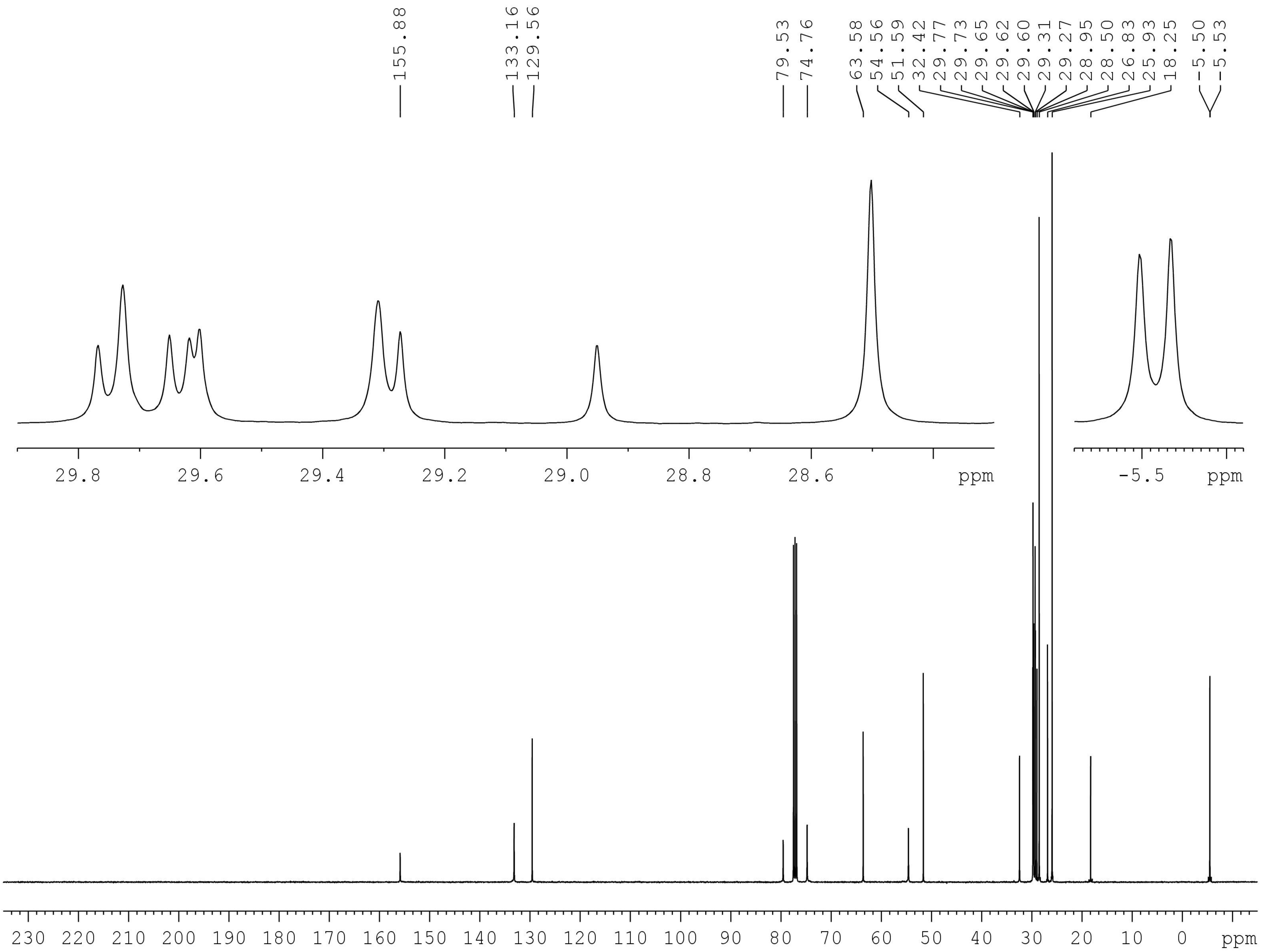

Supplementary Figure 12: ^{13}C NMR spectrum of **4** ($CDCl_3$, 100 MHz).

Supplementary Figure 13

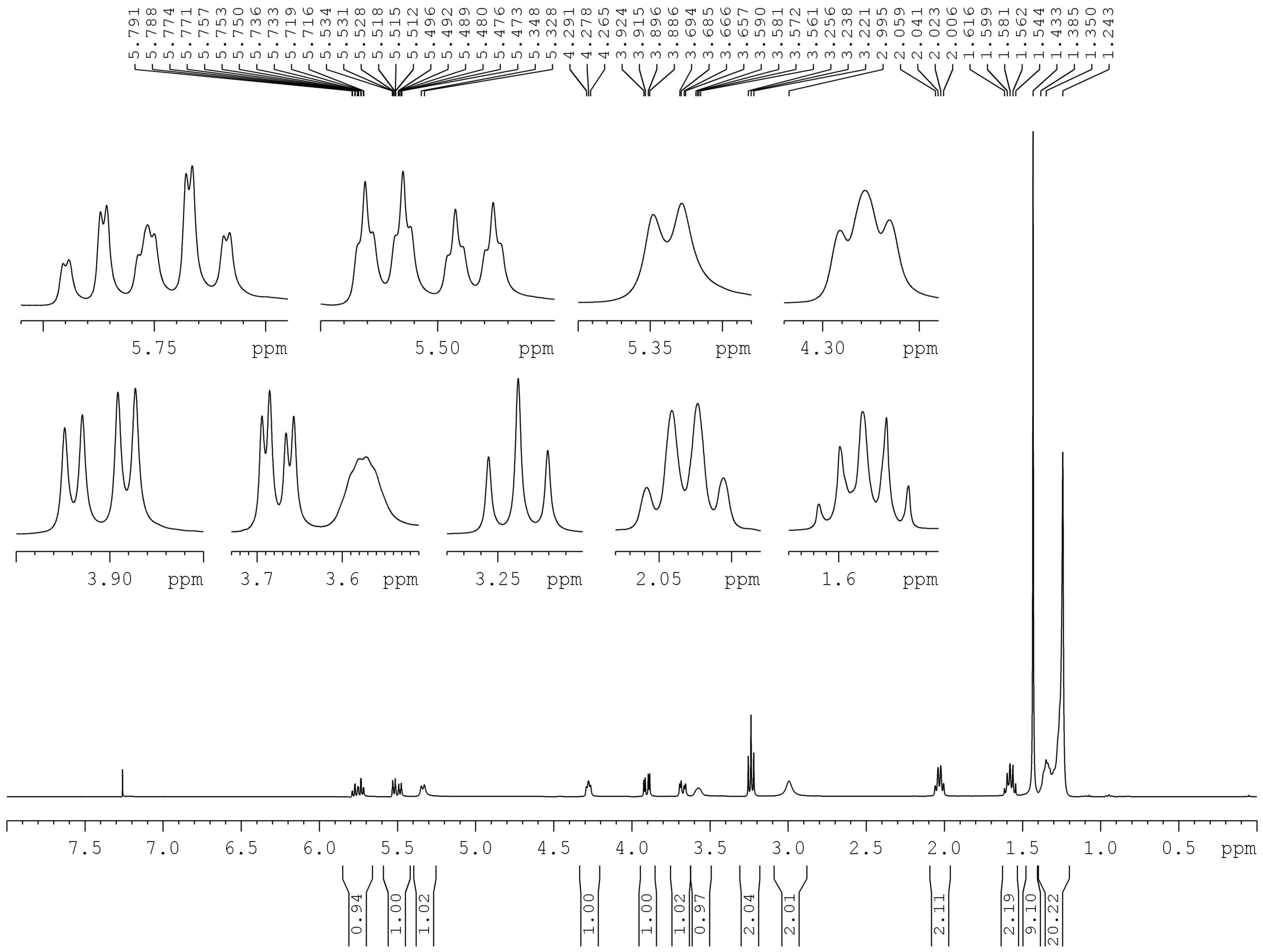

Supplementary Figure 13: ^{1}H NMR spectrum of **5** ($CDCl_3$, 400 MHz).

Supplementary Figure 14

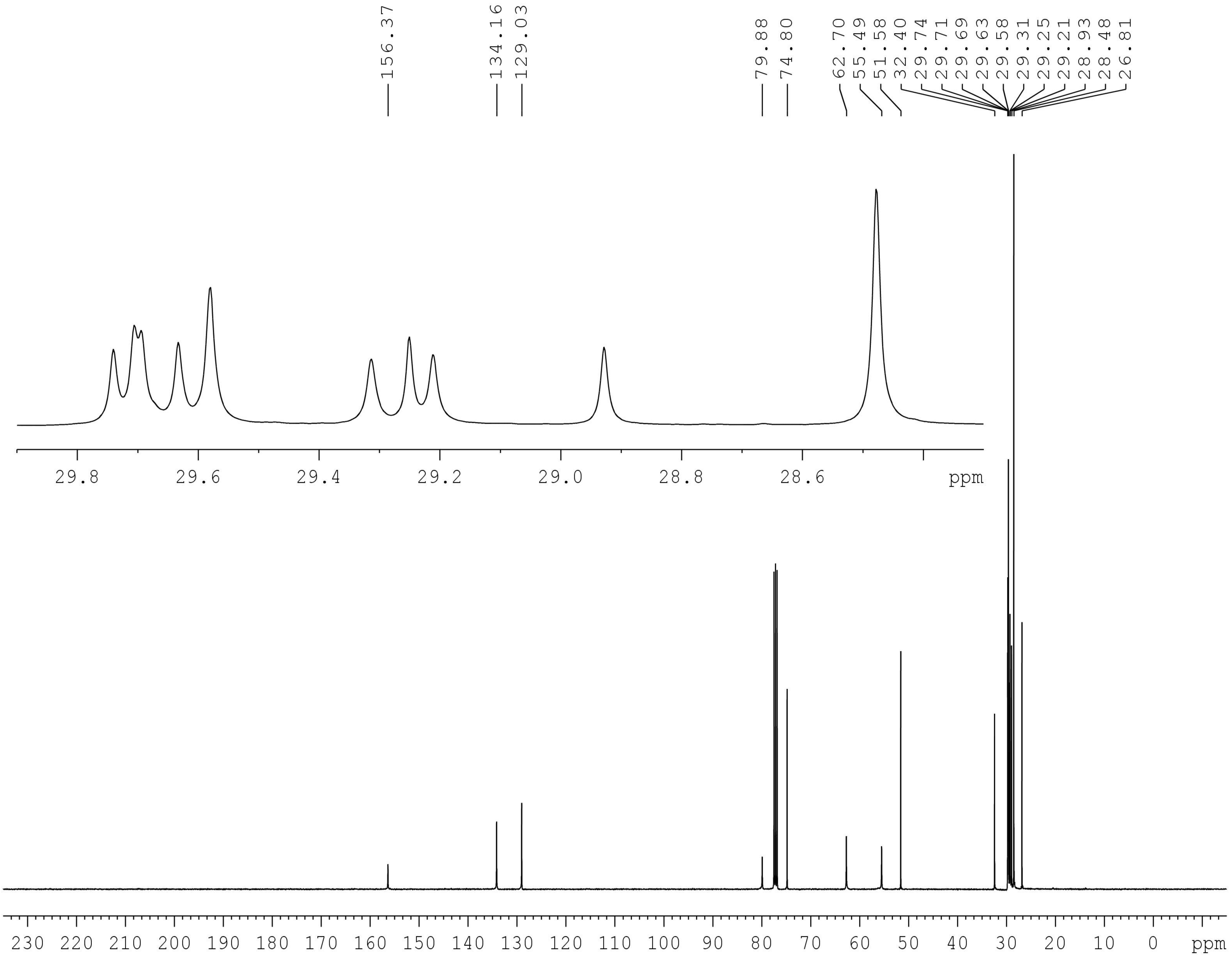

Supplementary Figure 14: ^{13}C NMR spectrum of **5** ($CDCl_3$, 100 MHz).

Supplementary Figure 15

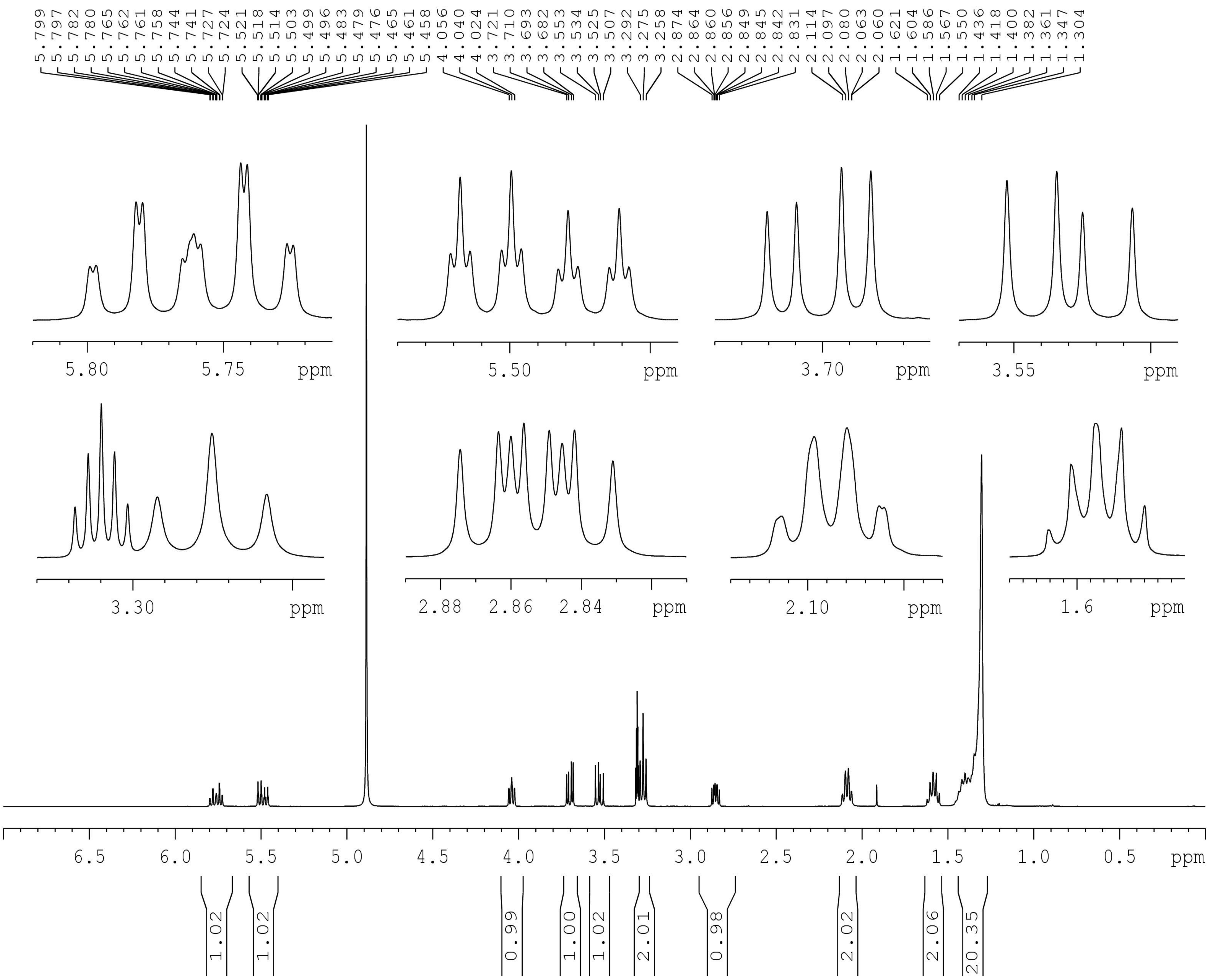

Supplementary Figure 15: ^{1}H NMR spectrum of **6** (CD_3OD, 400 MHz).

Supplementary Figure 16

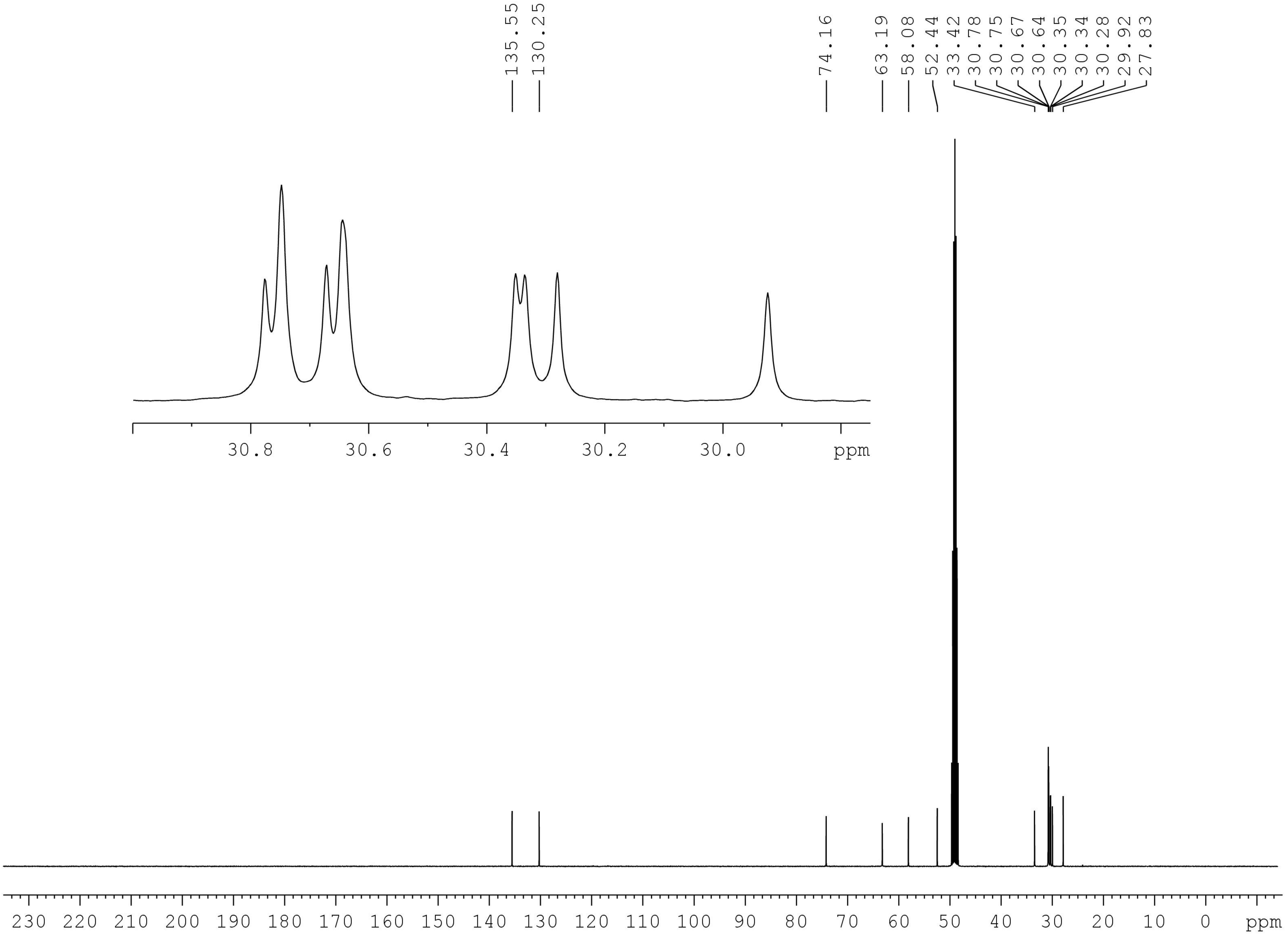

Supplementary Figure 16: ^{13}C NMR spectrum of **6** (CD_3OD, 100 MHz).

Supplementary Figure 17

Mass Spectrum SmartFormula Report

Analysis Info

Analysis Name	D:\Data\Spektren2016\2016_3206_SEI_24_01_1010.d	Acquisition Date	12/9/2016 9:37:06 AM
Method	automation_esi_tune_pos_mid_ja.m	Operator	J.Adelmann
Sample Name	2016_3206_SEI	Instrument	micrOTOF-Q III 8228888.20516
Comment	Fink Julian<br>JF021<br>6 pmol/ul in MeCN/CHCl3		

Acquisition Parameter

Source Type	ESI	Ion Polarity	Positive	Set Nebulizer	0.7 Bar
Focus	Not active	Set Funnel 1 RF	300.0 Vpp	Set Dry Heater	200 °C
Scan Begin	50 m/z	Set Funnel 2 RF	400.0 Vpp	Set Dry Gas	5.0 l/min
Scan End	3500 m/z	Set Hexapole RF	500.0 Vpp	Set Divert Valve	Source

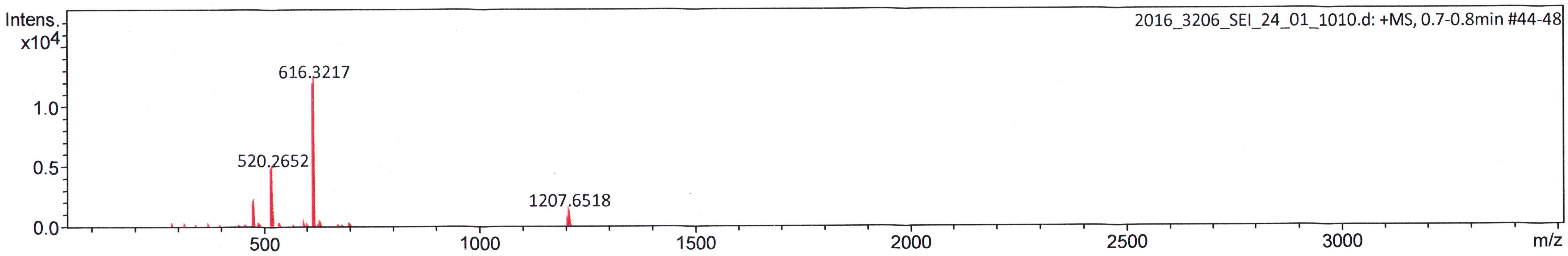

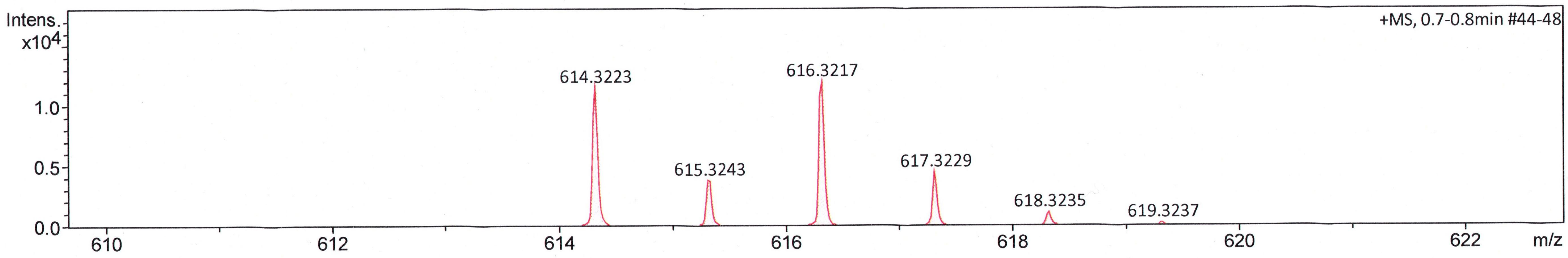

Meas. m/z	#	Ion Formula	m/z	err [ppm]	mSigma	# mSigma	Score	rdb	e⁻ Conf	N-Rule
614.3223	1	C29H58BrNNaO4Si	614.3211	-1.9	22.0	1	100.00	1.5	even	ok

Supplementary Figure 17: Mass spectrum of **3** (ESI^+).

Supplementary Figure 18

Mass Spectrum SmartFormula Report

**Analysis Info**		Acquisition Date	1/23/2017 1:30:13 PM
Analysis Name	D:\Data\Spektren2017\2017_0169_SEI.d		
Method	tune_wide.m	Operator	J.Adelmann
Sample Name	2017_0169_SEI	Instrument	micrOTOF-Q III 8228888.20516
Comment	Fink Julian<br>JF037<br>6pmol/uL in MeCN/CHCl3		

Acquisition Parameter

Source Type	ESI	Ion Polarity	Positive	Set Nebulizer	0.3 Bar
Focus	Not active	Set Funnel 1 RF	200.0 Vpp	Set Dry Heater	200 °C
Scan Begin	50 m/z	Set Funnel 2 RF	300.0 Vpp	Set Dry Gas	4.0 l/min
Scan End	4000 m/z	Set Hexapole RF	400.0 Vpp	Set Divert Valve	Source

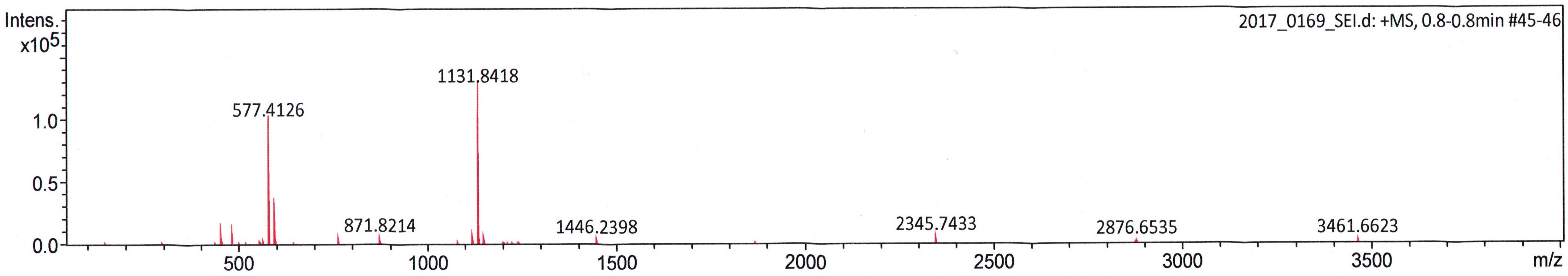

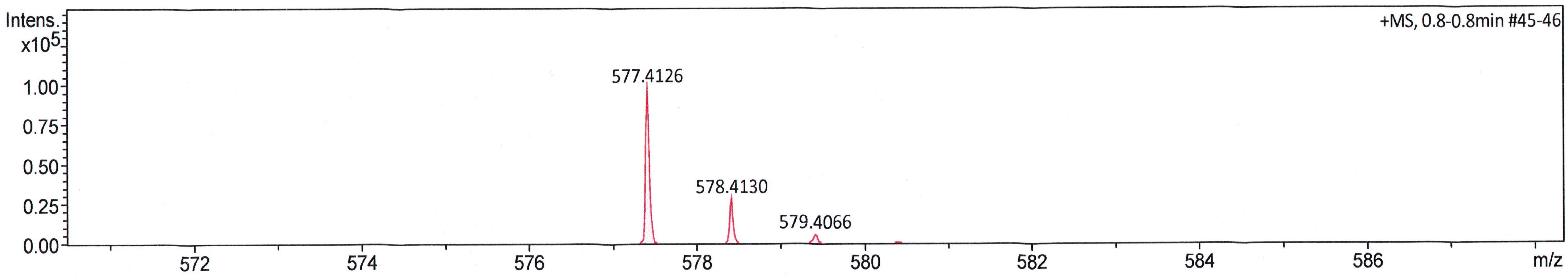

Meas. m/z	#	Ion Formula	m/z	err [ppm]	mSigma	# mSigma	Score	rdb	e^- Conf	N-Rule
577.4126	1	C29H58N4NaO4Si	577.4120	-1.1	55.6	1	100.00	3.5	even	ok

Supplementary Figure 18: Mass spectrum of **4** (ESI$^+$).

Supplementary Figure 19

Mass Spectrum SmartFormula Report

Analysis Info

Analysis Name	D:\Data\Spektren2017\2017_0337_SEI_90_01_1387.d	Acquisition Date	2/9/2017 2:33:17 PM
Method	automation_esi_tune_pos_mid_ja.m	Operator	J.Adelmann
Sample Name	2017_0337_SEI	Instrument	micrOTOF-Q III 8228888.20516
Comment	Fink Julian<br>JF047<br>4 pMol/uL in MeCN/CHCl3		

Acquisition Parameter

Source Type	ESI	Ion Polarity	Positive	Set Nebulizer	0.7 Bar
Focus	Not active	Set Funnel 1 RF	300.0 Vpp	Set Dry Heater	200 °C
Scan Begin	50 m/z	Set Funnel 2 RF	400.0 Vpp	Set Dry Gas	5.0 l/min
Scan End	3500 m/z	Set Hexapole RF	500.0 Vpp	Set Divert Valve	Source

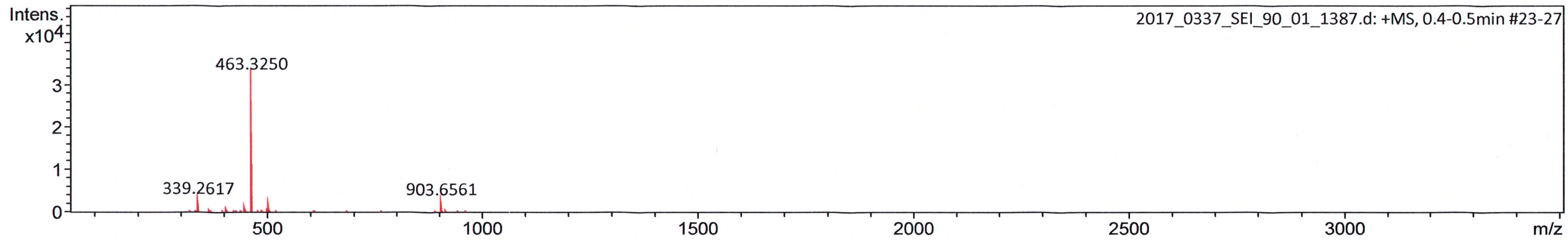

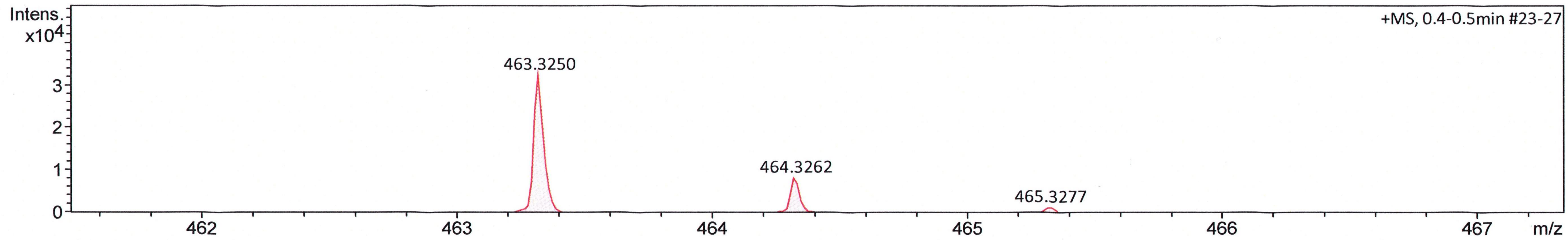

Meas. m/z	#	Ion Formula	m/z	err [ppm]	mSigma	# mSigma	Score	rdb	e⁻ Conf	N-Rule
463.3250	1	C23H44N4NaO4	463.3255	0.9	11.2	1	100.00	3.5	even	ok

Supplementary Figure 19: Mass spectrum of **5** (ESI$^+$).

Supplementary Figure 20

Mass Spectrum SmartFormula Report

Analysis Info

Analysis Name	D:\Data\Spektren2017\2017_0514_SEI_11_01_1441.d	Acquisition Date	2/16/2017 1:20:24 PM
Method	automation_esi_tune_pos_low_ja_meoh.m	Operator	J.Adelmann
Sample Name	2017_0514_SEI	Instrument	micrOTOF-Q III 8228888.20516
Comment	Fink Julian<br>JF052<br>3 pMol/uL in MeOH		

Acquisition Parameter

Source Type	ESI	Ion Polarity	Positive	Set Nebulizer	0.7 Bar
Focus	Not active	Set Funnel 1 RF	100.0 Vpp	Set Dry Heater	200 °C
Scan Begin	50 m/z	Set Funnel 2 RF	300.0 Vpp	Set Dry Gas	5.0 l/min
Scan End	5000 m/z	Set Hexapole RF	200.0 Vpp	Set Divert Valve	Source

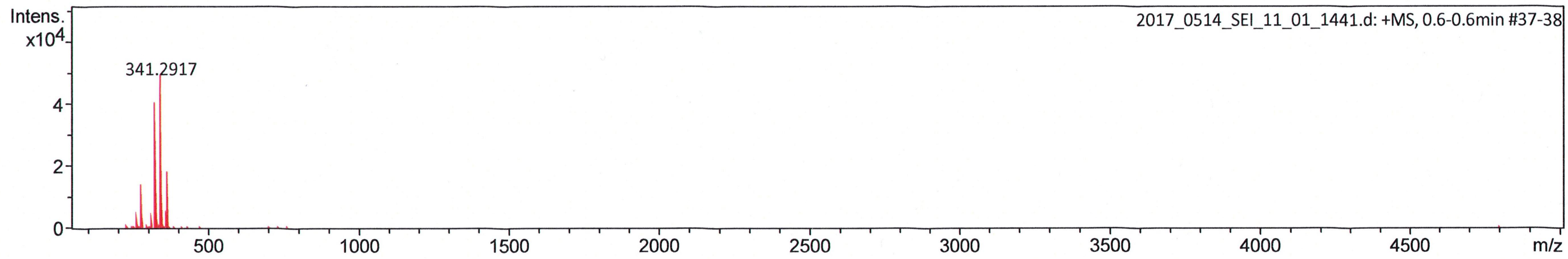

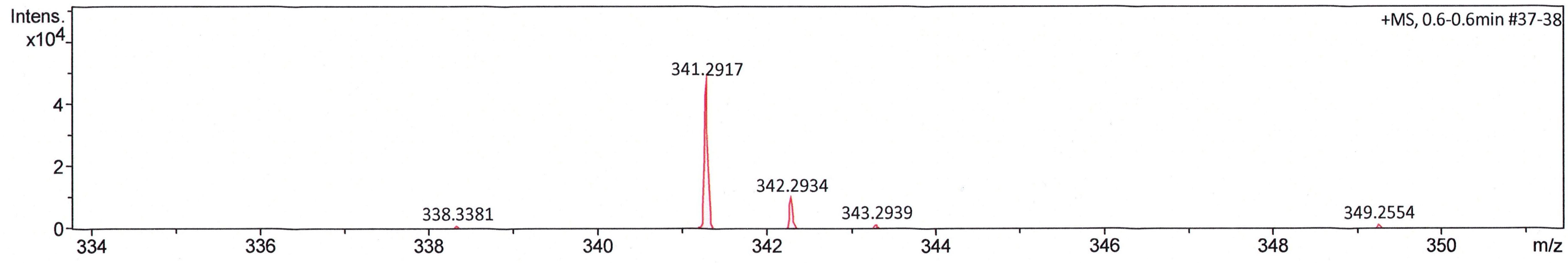

Meas. m/z	#	Ion Formula	m/z	err [ppm]	mSigma	# mSigma	Score	rdb	e⁻ Conf	N-Rule
341.2917	1	C18H37N4O2	341.2911	-1.8	2.1	1	100.00	2.5	even	ok

Supplementary Figure 20: Mass spectrum of **6** (ESI^+).

Supplementary Table 1

a

GROUP	RANK (NES)	NAME	SIZE	ES	NES	FDR q-val	FWER p-val	RANK AT MAX	LEADING EDGE
clathrin-coated membranes	1	GO_AP_TYPE_MEMBRANE_COAT_ADAPTOR_COMPLEX	37	0,6817025	2,139373	0,009	0	4769	tags=73%, list=20%, signal=91%
	2	GO_CLATHRIN_COATED_VESICLE	140	0,4177536	2,1109693	0,009	0,009	3360	tags=36%, list=14%, signal=42%
	6	GO_CLATHRIN_ADAPTOR_COMPLEX	27	0,6502399	2,0203426	0,018837286	0,063	4769	tags=70%, list=20%, signal=87%
	15	GO_CLATHRIN_COAT	45	0,6242246	1,87694	0,025962245	0,153	4769	tags=67%, list=20%, signal=83%
	16	GO_TRANS_GOLGI_NETWORK_TRANSPORT_VESICLE	26	0,50127023	1,8702269	0,025858812	0,17	3360	tags=42%, list=14%, signal=49%
	19	GO_COATED_VESICLE	205	0,33293936	1,8079635	0,03574674	0,243	3360	tags=29%, list=14%, signal=33%
	20	GO_CLATHRIN_COATED_VESICLE_MEMBRANE	66	0,42314196	1,8042084	0,03488713	0,243	3124	tags=36%, list=13%, signal=42%
	34	GO_COATED_MEMBRANE	81	0,5143869	1,724187	0,0398519	0,381	4845	tags=51%, list=20%, signal=63%
	46	GO_CLATHRIN_VESICLE_COAT	22	0,56756634	1,6978397	0,035762247	0,429	3019	tags=55%, list=12%, signal=62%
endocytosis	30	GO_PHAGOCYTIC_VESICLE	73	0,52179927	1,7359599	0,03921569	0,35	4086	tags=51%, list=17%, signal=61%
	37	GO_PHAGOCYTIC_VESICLE_MEMBRANE	46	0,53581667	1,7175857	0,03818942	0,388	4086	tags=50%, list=17%, signal=60%
	50	GO_ENDOCYTIC_VESICLE	223	0,34582347	1,6779151	0,041357946	0,499	3881	tags=33%, list=16%, signal=39%
early endosome	3	GO_EARLY_ENDOSOME	262	0,4225165	2,0901139	0,009000001	0,009	4259	tags=40%, list=18%, signal=48%
	5	GO_EARLY_ENDOSOME_MEMBRANE	90	0,45907736	2,0442317	0,017993936	0,048	4368	tags=44%, list=18%, signal=54%
	40	GO_RETROMER_COMPLEX	19	0,70777726	1,7091843	0,037392348	0,419	4075	tags=68%, list=17%, signal=82%
late endosome	4	GO_LATE_ENDOSOME_MEMBRANE	87	0,49950457	2,0850801	0,009	0,009	4528	tags=52%, list=19%, signal=63%
	7	GO_LATE_ENDOSOME	184	0,44429532	2,0017903	0,020909633	0,085	4704	tags=44%, list=19%, signal=54%
	8	GO_ENDOSOMAL_PART	370	0,42579743	1,9757125	0,023100177	0,085	4576	tags=44%, list=19%, signal=53%
	33	GO_RECYCLING_ENDOSOME	118	0,40409502	1,7256137	0,0396777	0,381	4368	tags=41%, list=18%, signal=49%
lysosome	9	GO_LYTIC_VACUOLE	463	0,4277387	1,9631885	0,021533486	0,085	4530	tags=44%, list=19%, signal=53%
	12	GO_LYTIC_VACUOLE_MEMBRANE	235	0,48414743	1,9161127	0,025993459	0,14	4362	tags=49%, list=18%, signal=59%
	26	GO_VACUOLAR_LUMEN	104	0,3773395	1,7634717	0,0366276	0,319	3524	tags=38%, list=15%, signal=45%
	27	GO_BLOC_COMPLEX	17	0,6160383	1,7550533	0,03736286	0,319	3791	tags=59%, list=16%, signal=70%
	38	GO_LYSOSOMAL_LUMEN	81	0,38517004	1,7134563	0,037687793	0,401	3471	tags=41%, list=14%, signal=47%
cell division	18	GO_REPLICATION_FORK	58	0,5975565	1,8338519	0,031645566	0,223	6436	tags=71%, list=26%, signal=96%
	39	GO_MIDBODY	116	0,35698095	1,7105753	0,03786879	0,419	5584	tags=46%, list=23%, signal=59%
	41	GO_REPLISOME	28	0,6371837	1,7077754	0,036902785	0,419	6765	tags=75%, list=28%, signal=104%
	42	GO_NUCLEAR_REPLICATION_FORK	38	0,57244664	1,7048541	0,03673616	0,429	6436	tags=68%, list=26%, signal=93%
	54	GO_SPINDLE	252	0,3613654	1,653535	0,048039164	0,579	5675	tags=42%, list=23%, signal=54%
chromosomal region	28	GO_CHROMOSOME_CENTROMERIC_REGION	158	0,44841677	1,7494394	0,03919173	0,35	5584	tags=46%, list=23%, signal=59%
	52	GO_CHROMOSOMAL_REGION	295	0,39017215	1,6661352	0,04389605	0,553	6047	tags=44%, list=25%, signal=58%
microtubule	14	GO_CENTRIOLE	86	0,45790482	1,8774872	0,027173832	0,153	4879	tags=43%, list=20%, signal=54%
	45	GO_MICROTUBULE_ORGANIZING_CENTER_PART	123	0,42793038	1,6979736	0,03635697	0,429	5643	tags=46%, list=23%, signal=60%
proton transport complex	11	GO_PROTON_TRANSPORTING_TWO_SECTOR_ATPASE_COMPLEX_CATALYTIC_DOMAIN	16	0,67587835	1,9239477	0,026597569	0,13	4100	tags=56%, list=17%, signal=68%
	13	GO_PROTON_TRANSPORTING_V_TYPE_ATPASE_COMPLEX	22	0,6414163	1,8885471	0,027705144	0,14	4100	tags=64%, list=17%, signal=76%
	17	GO_PROTON_TRANSPORTING_TWO_SECTOR_ATPASE_COMPLEX	42	0,58050895	1,8385583	0,030986838	0,206	4100	tags=52%, list=17%, signal=63%
mitochondria	22	GO_ORGANELLE_INNER_MEMBRANE	485	0,3901529	1,7873535	0,036679573	0,278	5738	tags=40%, list=24%, signal=51%
	29	GO_ORGANELLAR_RIBOSOME	70	0,48045403	1,7429737	0,038928393	0,35	5017	tags=47%, list=21%, signal=59%
	31	GO_MITOCHONDRIAL_MEMBRANE_PART	154	0,4452351	1,7304742	0,040072706	0,37	6149	tags=45%, list=25%, signal=60%
	32	GO_INNER_MITOCHONDRIAL_MEMBRANE_PROTEIN_COMPLEX	93	0,4993963	1,7265102	0,040178046	0,37	5710	tags=46%, list=24%, signal=60%
	35	GO_INTRINSIC_COMPONENT_OF_ORGANELLE_MEMBRANE	237	0,32457468	1,7241015	0,038970415	0,381	4674	tags=33%, list=19%, signal=41%
	43	GO_MITOCHONDRIAL_MATRIX	389	0,34331876	1,7011355	0,036091138	0,429	5166	tags=36%, list=21%, signal=45%
	47	GO_MITOCHONDRIAL_PROTEIN_COMPLEX	123	0,44945076	1,6955893	0,03665585	0,475	6139	tags=46%, list=25%, signal=61%
	49	GO_RESPIRATORY_CHAIN	75	0,49076676	1,6788516	0,0411818	0,499	5710	tags=43%, list=24%, signal=56%
	51	GO_INTRINSIC_COMPONENT_OF_MITOCHONDRIAL_MEMBRANE	44	0,46190116	1,6689262	0,04424508	0,535	4932	tags=43%, list=20%, signal=54%
	53	GO_INTRINSIC_COMPONENT_OF_MITOCHONDRIAL_INNER_MEMBRANE	17	0,5082864	1,6564015	0,047987744	0,561	2770	tags=41%, list=11%, signal=46%
ungrouped	10	GO_AXON_CYTOPLASM	31	0,50222677	1,9246219	0,028357327	0,13	3791	tags=42%, list=16%, signal=50%
	21	GO_IMMUNOLOGICAL_SYNAPSE	32	0,54109186	1,7903459	0,037997648	0,278	4488	tags=47%, list=18%, signal=57%
	23	GO_TRANS_GOLGI_NETWORK_MEMBRANE	64	0,49522012	1,7766278	0,037670236	0,305	3780	tags=44%, list=16%, signal=52%
	24	GO_MICROBODY	129	0,3392097	1,7741073	0,036906812	0,305	5560	tags=37%, list=23%, signal=48%
	25	GO_PIGMENT_GRANULE	99	0,41682222	1,7652943	0,0377327	0,319	3780	tags=34%, list=16%, signal=41%
	36	GO_AUTOPHAGOSOME	68	0,3550243	1,7179679	0,039000235	0,388	4494	tags=43%, list=19%, signal=52%
	44	GO_EXTRINSIC_COMPONENT_OF_ORGANELLE_MEMBRANE	22	0,46452084	1,6993158	0,035895374	0,429	5634	tags=50%, list=23%, signal=65%
	48	GO_PML_BODY	87	0,46760246	1,6898024	0,037775308	0,483	5943	tags=45%, list=24%, signal=59%
	55	GO_EXTRINSIC_COMPONENT_OF_CYTOPLASMIC_SIDE_OF_PLASMA_MEMBRANE	95	0,33212984	1,6519495	0,04801346	0,579	2920	tags=26%, list=12%, signal=30%

b

Group	RANK (NES)	NAME	SIZE	ES	NES	FDR q-val	FWER p-val	RANK AT MAX	LEADING EDGE
extracellular matrix	1	GO_EXTRACELLULAR_MATRIX	403	-0,6421122	-2,1020749	0,046640944	0,029	3660	tags=48%, list=15%, signal=55%
	2	GO_PROTEINACEOUS_EXTRACELLULAR_MATRIX	336	-0,65685314	-2,0959249	0,027820474	0,038	3660	tags=48%, list=15%, signal=55%
ER/Golgi	3	GO_ENDOPLASMIC_RETICULUM_LUMEN	184	-0,5300437	-1,9498274	0,04489303	0,11	3157	tags=38%, list=13%, signal=43%
	5	GO_GOLGI_LUMEN	82	-0,5789842	-1,8918775	0,046840988	0,181	3386	tags=35%, list=14%, signal=41%
anchoring junctions	6	GO_CELL_SUBSTRATE_JUNCTION	387	-0,38705605	-1,8837918	0,045543816	0,199	3782	tags=36%, list=16%, signal=42%
	9	GO_ANCHORING_JUNCTION	469	-0,39091176	-1,8360764	0,047074653	0,275	4155	tags=37%, list=17%, signal=44%
projection membranes	7	GO_LAMELLIPODIUM_MEMBRANE	18	-0,76415986	-1,88053	0,042221658	0,199	3290	tags=72%, list=14%, signal=83%
	10	GO_NEURON_PROJECTION_MEMBRANE	32	-0,61009735	-1,8091	0,04889716	0,285	3837	tags=50%, list=16%, signal=59%
	14	GO_DENDRITE_MEMBRANE	18	-0,57180417	-1,7932113	0,04320775	0,303	2095	tags=39%, list=9%, signal=43%
platelet alpha granules	4	GO_VESICLE_LUMEN	96	-0,5390744	-1,9249805	0,04230829	0,11	3943	tags=40%, list=16%, signal=47%
	12	GO_PLATELET_ALPHA_GRANULE_LUMEN	53	-0,5812689	-1,8022362	0,047483716	0,303	3943	tags=42%, list=16%, signal=49%
	13	GO_PLATELET_ALPHA_GRANULE	72	-0,4918661	-1,7938668	0,045219976	0,303	3943	tags=39%, list=16%, signal=46%
gap junction, connexion	8	GO_GAP_JUNCTION	28	-0,61969125	-1,8709532	0,041193422	0,222	3631	tags=39%, list=15%, signal=46%
	11	GO_CONNEXON_COMPLEX	18	-0,6039202	-1,8056597	0,046751294	0,285	3631	tags=39%, list=15%, signal=46%

Supplementary Table 1: Gene set enrichment analysis (GSEA)

a: GSEA report of the most enriched Gene Ontology CC gene sets (FDR < 0.05) in macrophages, ranked by their normalized enrichment score and grouped by function/compartment. **b**: GSEA report of the most enriched Gene Ontology CC gene sets (FDR < 0.05) in fibroblasts, ranked by their normalized enrichment score and grouped by function/compartment.

Supplementary Table 2

Gene	Name
Acaa1a	3-ketoacyl-CoA thiolase A, peroxisomal
Acaa1b	3-ketoacyl-CoA thiolase B, peroxisomal
Acaa2	3-ketoacyl-CoA thiolase, mitochondrial
Asah1	Acid ceramidase
Asah2	Neutral ceramidase
Chpt1	Choline Phosphotransferase 1
Degs1	Delta 4-Desaturase, Sphingolipid 1
Degs2	Delta 4-Desaturase, Sphingolipid 2
Far1	Fatty Acyl-CoA Reductase 1
Far2	Fatty Acyl-CoA Reductase 2
Fdft1	Farnesyl-diphosphate farnesyltransferase 1
Fdps	farnesyl diphosphate synthase
Ggps1	Geranylgeranyl Diphosphate Synthase 1
Gk2	Glycerol Kinase 2
Gk5	Glycerol Kinase 5
Gnpat	Glyceronephosphate O-Acyltransferase
Gpd1	Glycerol-3-Phosphate Dehydrogenase 1
Gpd1l	Glycerol-3-Phosphate Dehydrogenase 1 Like
Gpd2	Glycerol-3-Phosphate Dehydrogenase 2
Hmgcr	3-Hydroxy-3-Methylglutaryl-CoA Reductase
Hmgcs1	3-Hydroxy-3-Methylglutaryl-CoA Synthase 1
Hmgcs2	3-Hydroxy-3-Methylglutaryl-CoA Synthase 2
Idi1	Isopentenyl-Diphosphate Delta Isomerase 1
Idi2	Isopentenyl-Diphosphate Delta Isomerase 2
Kdsr	3-Ketodihydrosphingosine Reductase
Lass2	Ceramide Synthase 2
Lass3	Ceramide Synthase 3
Lass4	Ceramide Synthase 4
Lass5	Ceramide Synthase 5
Lass6	Ceramide Synthase 6
Mvk	Mevalonate Kinase
Pcyt1a	Phosphate Cytidylyltransferase 1, Choline, Alpha
Pcyt1b	Phosphate Cytidylyltransferase 1, Choline, Beta
Pcyt2	Ethanolamine-phosphate cytidylyltransferase
Pemt	Phosphatidylethanolamine N-Methyltransferase
Pisd	Phosphatidylserine Decarboxylase
Plcb1	Phospholipase C Beta 1
Plcb2	Phospholipase C Beta 2
Plcb3	Phospholipase C Beta 3
Plcb4	Phospholipase C Beta 4
Plcg1	Phospholipase C Gamma 1
Plcg2	Phospholipase C Gamma 2
Plcl1	Phospholipase C Like 1 (Inactive)
Plcl2	Phospholipase C Like 2
Pmvk	Phosphomevalonate Kinase
Ptdss1	Phosphatidylserine Synthase 1
Ptdss2	Phosphatidylserine Synthase 2
Sgms1	Sphingomyelin Synthase 1
Sgms2	Sphingomyelin Synthase 2
Sgpl1	Sphingosine-1-Phosphate Lyase 1
Sgpp1	Sphingosine-1-Phosphate Phosphatase 1
Sgpp2	Sphingosine-1-Phosphate Phosphatase 2
Smpd1	Sphingomyelin Phosphodiesterase 1
Smpd2	Sphingomyelin Phosphodiesterase 2
Smpd3	Sphingomyelin Phosphodiesterase 3
Smpd4	Sphingomyelin Phosphodiesterase 4
Sphk1	Sphingosine Kinase 1
Sphk2	Sphingosine Kinase 2
Sptlc1	Serine Palmitoyltransferase Long Chain Base Subunit 1
Sptlc2	Serine Palmitoyltransferase Long Chain Base Subunit 2
Sptlc3	Serine Palmitoyltransferase Long Chain Base Subunit 3
Sqle	Squalene Epoxidase
Tpi1	Triosephosphate Isomerase 1

Supplementary Table 2: Membrane-modulating proteins in macrophages vs fibroblasts
Membrane-modulating proteins from the families of sphingolipids (including gangliosides), cholesterol and phosphatidylcholine which were considered for simplified comparison of macrophages and fibroblasts.
